# Metabolic hallmarks of regulated cell death

**DOI:** 10.1038/s41422-026-01306-9

**Published:** 2026-10-20

**Authors:** Rui Kang, Xin Chen, Guido Kroemer, Daolin Tang

**Affiliations:** 1 Department of Surgery, UT Southwestern Medical Center, Dallas, Texas USA; 2 Guangzhou Municipal and Guangdong Provincial Key Laboratory of Protein Modification and Disease, School of Basic Medical Sciences, Guangzhou Medical University, Guangzhou, Guangdong, China; 3 Key Laboratory of Biological Targeting Diagnosis, Therapy and Rehabilitation of Guangdong Higher Education Institutes, The Fifth Affiliated Hospital, Guangzhou Medical University, Guangzhou, Guangdong, China; 4 Centre de Recherche des Cordeliers, Université Paris Cité, Sorbonne Université, Inserm, Paris, France; 5 Institut Gustave Roussy, Université Paris-Saclay, INSERM US23/CNRS UAR 3655, Metabolomics and Cell Biology Platforms, UMS AMMICa, Villejuif Cedex, France; 6 Institut du Cancer Paris CARPEM, Department of Biology, Hôpital Européen Georges-Pompidou, AP-HP, Paris, France; 7 Centre de Recherche des Cordeliers, Équipe labellisée par la Ligue contre le cancer, Paris, France

**Keywords:** Cell death, Cancer metabolism

## Abstract

Regulated cell death (RCD) is characterized by distinct molecular execution mechanisms, but its initiation, progression, and biological consequences are increasingly recognized as being shaped by cellular metabolism. Bioenergetic state, redox homeostasis, nutrient availability, metal ion metabolism, inflammatory metabolism, and organelle function collectively determine cellular susceptibility to death and influence the activation and execution of diverse RCD programs. In this review, we synthesize the metabolic hallmarks of established, independently validated, and recently proposed forms of RCD, highlighting both their mechanistic diversity and their extensive metabolic convergence. We then examine the endogenous mechanisms that maintain metabolic homeostasis and discuss how their disruption reshapes cell death responses in human disease and therapeutic contexts. Together, these insights provide a guide to  the metabolic landscape of RCD and reveal metabolism not only as a modifier of cell death, but as a fundamental determinant of when, how, and with what consequences cells die. We conclude by discussing key conceptual and mechanistic challenges and emerging opportunities for therapeutically exploiting the metabolic control of RCD.

## Introduction

Cell death is a fundamental biological process that shapes development, maintains tissue homeostasis, and eliminates damaged, senescent, or infected cells. Dysregulated cell death underlies many human diseases, including cancer, neurodegeneration, ischemic injury, and chronic inflammation. Historically, cell death was classified primarily on the basis of morphological features. This effort culminated in the recognition of apoptosis as a form of programmed cellular demise characterized by nuclear shrinkage, chromatin condensation, and apoptotic body formation.^1^ Advances in biochemistry and molecular biology subsequently transformed the field into a pathway-centered discipline. Distinct regulated cell death (RCD) modalities came to be defined by specific execution mechanisms and signaling modules.^2–4^ To standardize this rapidly expanding field, the Nomenclature Committee on Cell Death (NCCD) established classifications based on biochemical degradation pathways rather than morphology alone.^5,6^ These consensus criteria have provided a common conceptual and experimental foundation for modern cell death research.

**Fig. 1:**
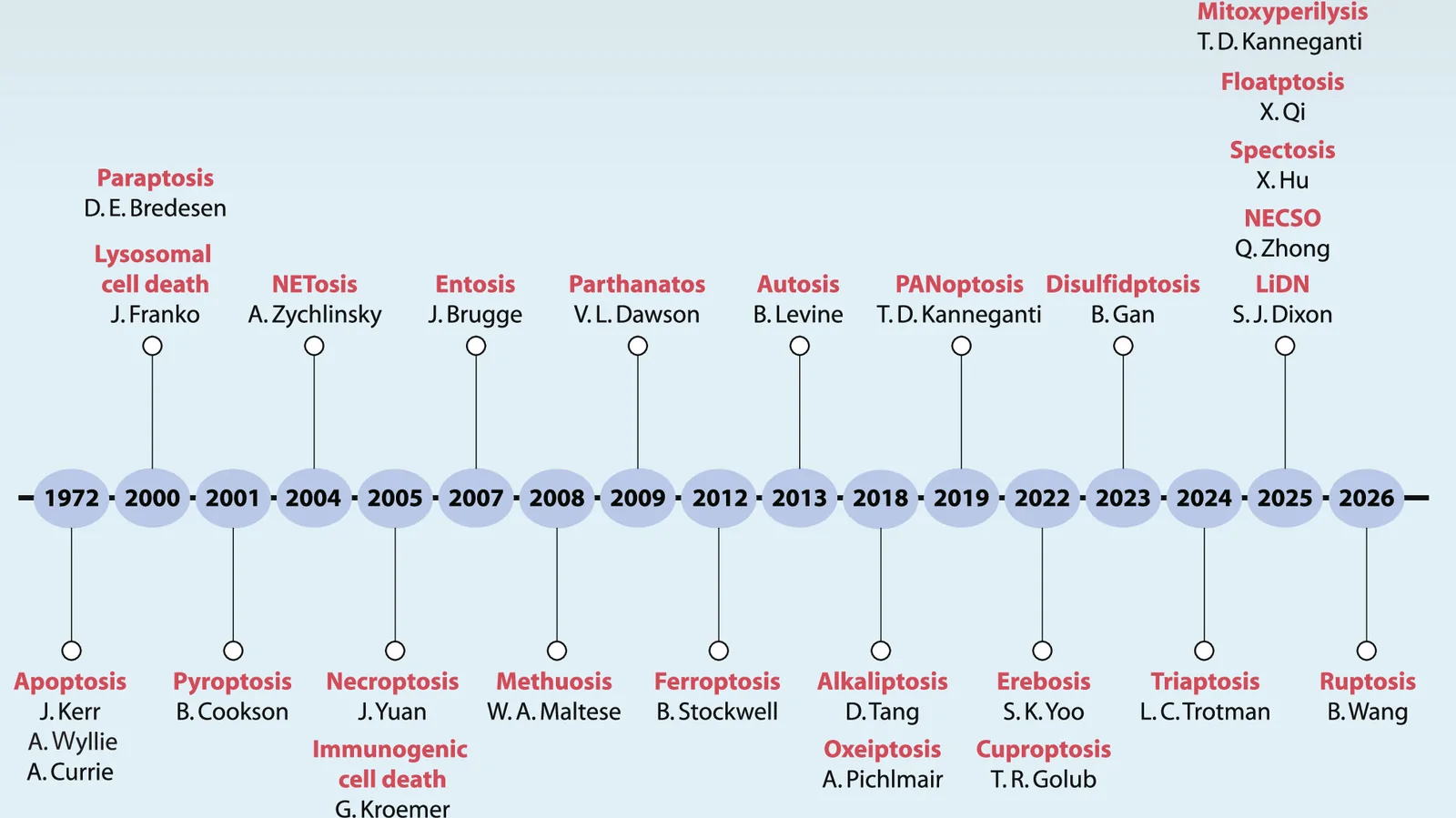
Timeline of major discoveries in cell death research. This timeline summarizes the initial description or naming of major cell death modalities reported from 1972 to 2026. These discoveries have progressively expanded the repertoire of recognized cell death programs and revealed increasing mechanistic diversity in the regulation of cellular demise.

Major forms of RCD, including apoptosis, necroptosis, pyroptosis, and ferroptosis, are now widely accepted, whereas several additional modalities have been proposed more recently, with various degrees of experimental support and community consensus (Fig. 1). Each death modality is defined by characteristic execution mechanisms, such as caspase activation in apoptosis,^7,8^ mixed lineage kinase domain-like pseudokinase (MLKL)-mediated membrane disruption in necroptosis,^9^ gasdermin pore formation in pyroptosis,^10–13^ and iron-dependent lethal phospholipid peroxidation in ferroptosis.^14^ Although these execution pathways are mechanistically distinct, increasing evidence indicates that they are all profoundly influenced by cellular metabolism.^15–17^ Bioenergetic status, redox homeostasis, nutrient availability, metal ion metabolism, lipid remodeling, and organelle function not only determine cellular adaptation to stress but also influence the initiation, progression, and biological consequences of many forms of RCD.^18^

In this review, we provide a comprehensive synthesis of the metabolic hallmarks of RCD. We summarize the metabolic alterations associated with each death modality, discuss the endogenous mechanisms that maintain metabolic homeostasis and shape cellular susceptibility to death, and examine how metabolic regulation contributes to cancer, neurodegeneration, infection, inflammatory diseases, and therapeutic responses. By integrating current evidence across diverse forms of RCD, we provide an updated perspective on the metabolic landscape of RCD, highlighting shared principles, defining features, and key questions that will guide future research.

## Metabolic principles governing RCD

**Fig. 2:**
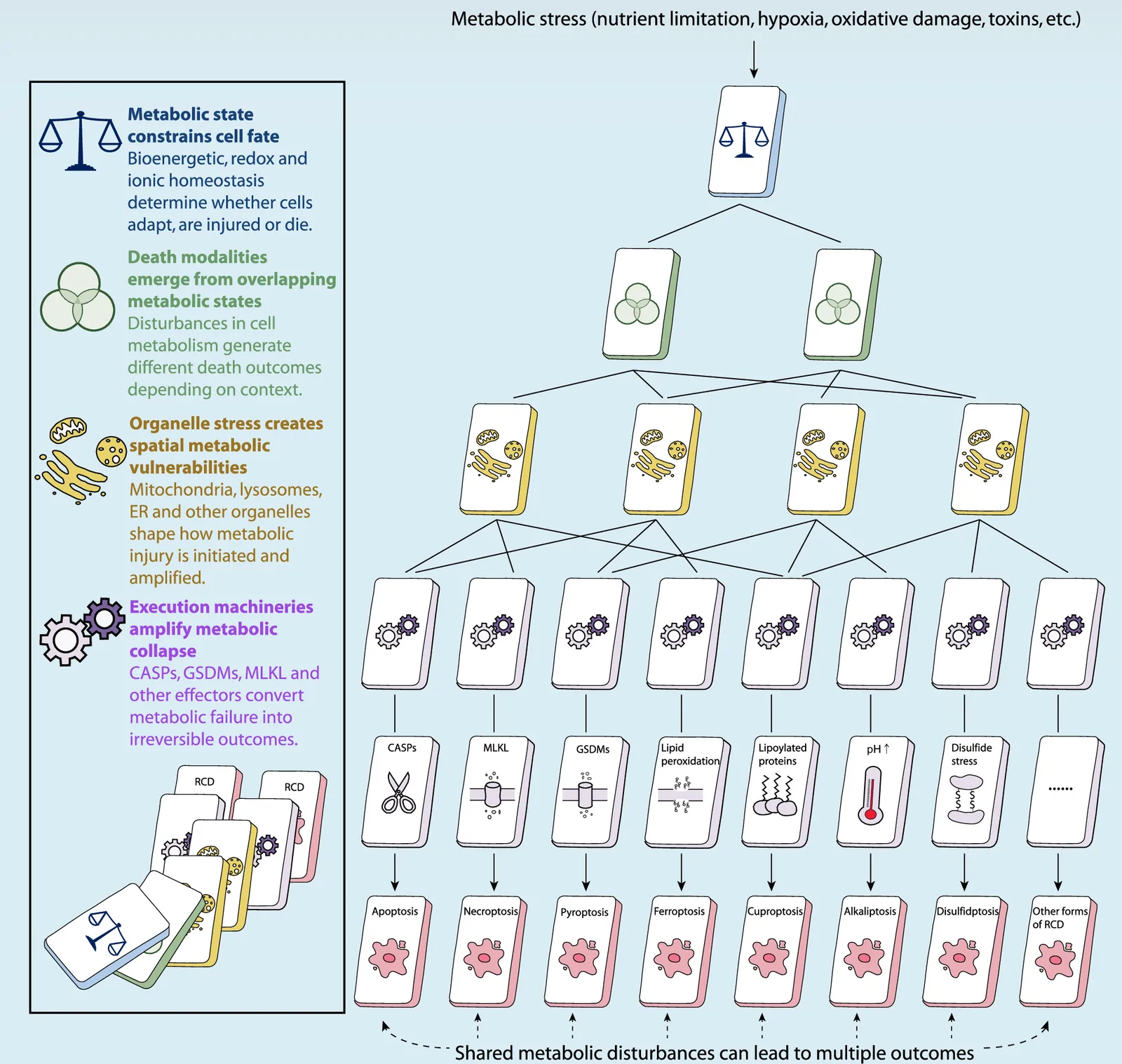
A metabolism-centered model of regulated cell death. Diverse forms of metabolic stress, including nutrient limitation, hypoxia, oxidative damage, and toxic insults, perturb cellular metabolic homeostasis. Cellular fate is determined by the ability to maintain bioenergetic capacity, redox balance, and ionic homeostasis. Shared metabolic disturbances converge on overlapping states of metabolic vulnerability and are further shaped by organelle-specific dysfunction involving mitochondria, lysosomes, the endoplasmic reticulum (ER), and other intracellular compartments. These metabolic perturbations engage distinct execution mechanisms, including caspases (CASPs), mixed lineage kinase domain-like pseudokinase (MLKL), gasdermins (GSDMs), lipid peroxidation, lipoylated protein aggregation, intracellular alkalinization, and disulfide stress, resulting in apoptosis, necroptosis, pyroptosis, ferroptosis, cuproptosis, alkaliptosis, disulfidptosis, and other forms of regulated cell death. This model demonstrates that common metabolic disturbances can generate multiple death outcomes depending on cellular context, adaptive capacity, and effector engagement.

Rather than functioning as fully isolated pathways, RCD programs often emerge from interconnected metabolic states that evolve as cells adapt, or fail to adapt, to stress. This perspective shifts attention away from individual execution modules and toward the metabolic conditions that determine whether stressed cells recover, progress toward injury, or ultimately undergo irreversible demise. Several common principles emerge, as discussed below (Fig. 2).

### Metabolic state constrains cell fate

The ability of cells to survive stress depends on the coordinated maintenance of bioenergetic, redox, and ionic homeostasis rather than adenosine triphosphate (ATP) production alone. Once these systems become uncoupled, adaptive responses fail, giving way to irreversible injury.

Bioenergetic collapse represents one of the clearest examples of this principle. Severe ATP depletion compromises membrane transport, proteostasis, and biosynthetic maintenance, thereby destabilizing fundamental cellular functions.^19^ Nonetheless, RCD does not simply arise from catastrophic energy exhaustion. In many settings, cells initiate death programs while substantial metabolic activity persists. Apoptotic cells, for example, retain sufficient ATP to support caspase activation and membrane remodeling, whereas severe energetic failure often favors lytic forms of cellular disintegration.^8,20^ The mode of death therefore reflects the metabolic state of the cell at the very moment that execution pathways engage.

Nicotinamide adenine dinucleotide (NAD^+^) depletion further illustrates how metabolic imbalance constrains cell fate. Hyperactivation of poly(ADP-ribose) polymerase 1 (PARP1) following extensive DNA damage consumes NAD^+^, impairing both glycolysis and mitochondrial respiration,^21,22^ and thus driving the energetic collapse characteristic of parthanatos.^23^ Similarly, sustained sodium or calcium influx imposes excessive energetic demands on ATP-dependent ion pumps, creating self-reinforcing cycles of mitochondrial dysfunction and metabolic exhaustion.^24,25^

Metabolic constraints are not just passive consequences of injury. They actively shape death susceptibility. Cells with limited antioxidant capacity, restricted metabolic flexibility, or high mitochondrial dependence often exhibit selective vulnerability to metabolic stress. Metabolism therefore defines the cellular conditions that permit or restrict the engagement of regulated death pathways.

### Death modalities emerge from overlapping metabolic states

Traditional classification systems tend to present RCD modalities as distinct biochemical degradation programs. However, the metabolic features that underlie these pathways overlap extensively. Mitochondrial dysfunction, oxidative stress, phospholipid remodeling, and nutrient limitation recur across many apparently separate death programs.

Ferroptosis illustrates this convergence particularly well. Although operationally defined by iron-dependent lipid peroxidation, ferroptosis involves an iron-independent redox imbalance,^26–28^ altered mitochondrial metabolism,^29–31^ nicotinamide adenine dinucleotide phosphate (NADPH) depletion,^32,33^ and phospholipid remodeling.^34–36^ In ferroptosis, lipid peroxidation functions simultaneously as a metabolic stress, signaling event, and lethal effector process. Necroptosis is similarly associated with mitochondrial generation of reactive oxygen species (ROS) and energetic deterioration,^37,38^ whereas pyroptosis emerges within inflammatory states characterized by excessive glycolysis and altered metabolite signaling.^39,40^

These overlaps suggest that at least some differences between death modalities may reflect variations in cellular and metabolic context rather than entirely separate biological programs. Distinct execution phenotypes can emerge from related metabolic disturbances, depending on nutrient availability, stress intensity, cell lineage, and tissue microenvironment. Inflammatory stress, for example, can engage overlapping pyroptotic, necroptotic, and ferroptosis-associated features.^41,42^

Metabolic transitions during cell death are rarely static. Bioenergetic failure can amplify oxidative injury, redox imbalance can impair organelle function, and accumulation of signaling metabolites can amplify the disruption of cellular homeostasis.^43,44^ Different execution pathways may therefore represent alternative trajectories through partially shared metabolic states.

This perspective does not disregard the mechanistic distinctions between death modalities but instead places these distinctions within a broader metabolic continuum. Death phenotypes may therefore depend less on isolated signaling modules than on the means by which execution machineries engage with specific metabolic constraints.

### Organelle stress creates spatial metabolic vulnerabilities

At the subcellular level, metabolic dysfunction evolves in a spatially organized context because organelles have distinct metabolic functions and stress-buffering capacities. Therefore, the primary organelle affected by metabolic injury influences how death signals are ignited, amplified, and propagated.

Mitochondria occupy a central position in RCD owing to their key roles in energy production, ROS generation, iron metabolism, and the integration of cellular stress signals. Mitochondrial dysfunction regulates multiple death pathways, although the outcome depends on the type and intensity of stress. Loss of mitochondrial membrane integrity can trigger the release of cytochrome c (CYCS) and other mitochondrial factors that initiate apoptotic signaling and broader cellular stress responses,^45–49^ whereas disruption of mitochondrial redox homeostasis drives oxidative damage and further destabilizes cellular metabolism.^30,50,51^ In many settings, mitochondria may represent an early point of convergence for metabolic stress signals.

Lysosomes are another major axis of metabolic integration. In addition to their degradative functions, lysosomes regulate nutrient recycling, lipid turnover, intracellular metal storage, and autophagic flux.^52^ Disruption of lysosomal integrity can compromise intracellular compartmentalization, allowing hydrolytic enzymes and stored metabolites to influence cellular stress responses and injury propagation, ultimately contributing to lysosome-dependent cell death.^53,54^ Lysosome-associated autophagic responses further illustrate the dual nature of metabolic stress adaptation. Although initially protective, sustained activation of autophagy can become maladaptive and contribute to cellular dysfunction and death, a process termed autophagy-dependent cell death.^55,56^

The endoplasmic reticulum (ER) coordinates protein folding, lipid synthesis, and calcium homeostasis. Persistent ER stress disrupts proteostasis and organelle communication, thereby amplifying mitochondrial dysfunction and inflammatory signaling.^57,58^ Endosomal and vesicular systems also shape metabolic dependency by regulating nutrient uptake, membrane turnover, and intracellular trafficking. Defects in these pathways contribute to vacuolization-associated forms of cell death such as paraptosis, which is characterized by the extensive accumulation of cytoplasmic vacuoles and the progressive loss of intracellular organization.^59^

Communication between organelles also influences the spatial organization of metabolic stress. Membrane contact sites linking the ER, mitochondria, and lysosomes coordinate calcium flux, lipid trafficking, and metabolic signaling under both physiological and stress conditions. Disruption of these interactions amplifies mitochondrial dysfunction, alters redox balance, and facilitates the propagation of metabolic injury across intracellular compartments.^60^ Metabolic stress therefore spreads not only through local organelle damage but also through failure of inter-organelle coordination.

Other organelles also contribute to the metabolic organization that influences cellular responses to stress and death. For example, peroxisomes,^61,62^ lipid droplets,^63,64^ the Golgi apparatus,^65,66^ and the nucleus^67,68^ participate in redox regulation, lipid handling, membrane trafficking, and stress signaling.

Organelle vulnerability is therefore likely to be heterogeneous and context-dependent rather than uniformly distributed across intracellular compartments. Distinct organelles may act as major nodes for the perception, integration, and propagation of metabolic stress, depending on tissue context, nutrient availability, and pathological conditions.

### Execution machineries amplify metabolic collapse

Canonical execution proteins are essential mediators of RCD phenotypes. Caspases, gasdermins, MLKL, and related effectors drive membrane disruption and irreversible structural disassembly. However, accumulating evidence suggests that their activation is frequently influenced by the metabolic conditions that determine whether cells adapt to stress or progress toward death.

Metabolic perturbations influence the activation threshold, efficiency, and phenotypic consequences of execution pathways. Thus, ATP availability is critical for apoptosome assembly and postmitochondrial caspase activation^8,45^; ROS modulate inflammasome signaling and kinase activity^69,70^; and membrane lipid composition conditions ferroptotic sensitivity.^34–36^ In many contexts, execution pathways amplify cellular injury that has already exceeded the metabolic buffering capacity. For example, permeabilization of cellular or organellar membranes disrupts ion gradients and metabolite retention^11,71^; mitochondrial injury can amplify a looming bioenergetic or redox collapse^51^; and inflammatory signaling also enhances oxidative stress and metabolic dysfunction.^40,69^ As these feedback loops intensify, cells inexorably shift from attempted stress adaptation to irreversible structural collapse.

## Metabolic features of cell death modalities

To facilitate discussion of the rapidly expanding landscape of RCD, the modalities discussed below are organized according to their current level of experimental support and community consensus. Accordingly, we distinguish among established forms, independently validated forms, and recently proposed forms of RCD. Established forms are supported by broad mechanistic consensus and are widely accepted within the field. Independently validated forms have been reproduced by multiple independent laboratories and are supported by substantial experimental evidence, although their physiological scope, defining mechanisms, and/or consensus criteria continue to evolve. Recently proposed forms are currently supported primarily by the original reports and await broader independent validation.

Although these modalities differ in their defining execution mechanisms, they are associated with recurrent metabolic perturbations involving bioenergetics, redox homeostasis, lipid metabolism, metal ion handling, inflammatory metabolism, and organelle function. These metabolic processes frequently overlap, and individual forms of RCD may exhibit multiple metabolic hallmarks depending on the cellular context, stress conditions, and disease setting. The following sections summarize the metabolic features of each RCD modality within the context of these shared metabolic themes, emphasizing both their characteristic metabolic alterations and their mechanistic distinctions (Table 1).

**Table 1:** Mechanisms and metabolic hallmarks of RCD

RCD modality	Evidence status	Key mechanism	Major metabolic hallmarks
Apoptosis	Established	Caspase activation following intrinsic/extrinsic signaling	ATP dependence; mitochondrial metabolism
Pyroptosis	Established	Inflammasome-gasdermin pore formation	Glycolysis; mitochondrial ROS; immunometabolism
Necroptosis	Established	RIPK1/RIPK3-MLKL activation	Ion imbalance; ATP consumption; mitochondrial ROS
Ferroptosis	Established	Iron-dependent phospholipid peroxidation	Iron metabolism; lipid peroxidation; GSH/GPX4; NADPH
Lysosome-dependent cell death	Independently validated	Lysosomal membrane permeabilization	Lysosomal metabolism; cholesterol; autophagy
Paraptosis	Independently validated	ER/mitochondrial vacuolization	ER stress; Ca^2+^ homeostasis; mitochondrial dysfunction
NETosis	Independently validated	NET release	Glycolysis; NADPH oxidase; ROS
Entotic cell death	Independently validated	Cell-in-cell engulfment	Nutrient stress; AMPK signaling
Methuosis	Independently validated	Macropinosome accumulation	Endosomal trafficking; nutrient scavenging
Parthanatos	Independently validated	PARP1-AIFM1 signaling	NAD^+^ depletion; ATP loss
Autophagy-dependent cell death	Independently validated	ATG-dependent autophagy	Nutrient sensing; autophagic metabolism
Oxeiptosis	Independently validated	KEAP1-PGAM5-AIFM1 signaling	ROS metabolism
Alkaliptosis	Independently validated	Intracellular alkalinization	pH homeostasis; lysosomal function
PANoptosis	Independently validated	Integrated pyroptotic, apoptotic, and necroptotic machinery	Immunometabolism; mitochondrial stress
Cuproptosis	Independently validated	Copper-induced lipoylated protein aggregation	Copper homeostasis; mitochondrial metabolism
Disulfidptosis	Independently validated	Actin cytoskeleton collapse	Thiol metabolism; NADPH depletion
Triaptosis	Recently proposed	PI3P-dependent endosomal disruption	Endosomal metabolism
Necrosis by sodium overload (NECSO)	Recently proposed	Sustained Na^+^ overload	Na^+^ homeostasis; ATP depletion
Lipid-dependent necrosis (LiDN)	Recently proposed	CIL56/tegavivint-induced necrosis	Fatty acid synthesis; protein palmitoylation
Mitoxyperilysis	Recently proposed	Localized mitochondrial ROS injury	Mitochondrial ROS; GSH depletion
Spectosis	Recently proposed	miniNLRP3-CASP8-mediated erythrocyte lysis	Glycolysis; redox homeostasis
Erebosis	Recently proposed	Progressive epithelial cell elimination	Progressive metabolic decline
Floatptosis	Recently proposed	Infection-associated endolysosomal vacuolization	Endolysosomal metabolism
Ruptosis	Recently proposed	Activin-induced membrane rupture	ER Ca^2+^ signaling

## Established forms of RCD

### Apoptosis

**Fig. 3:**
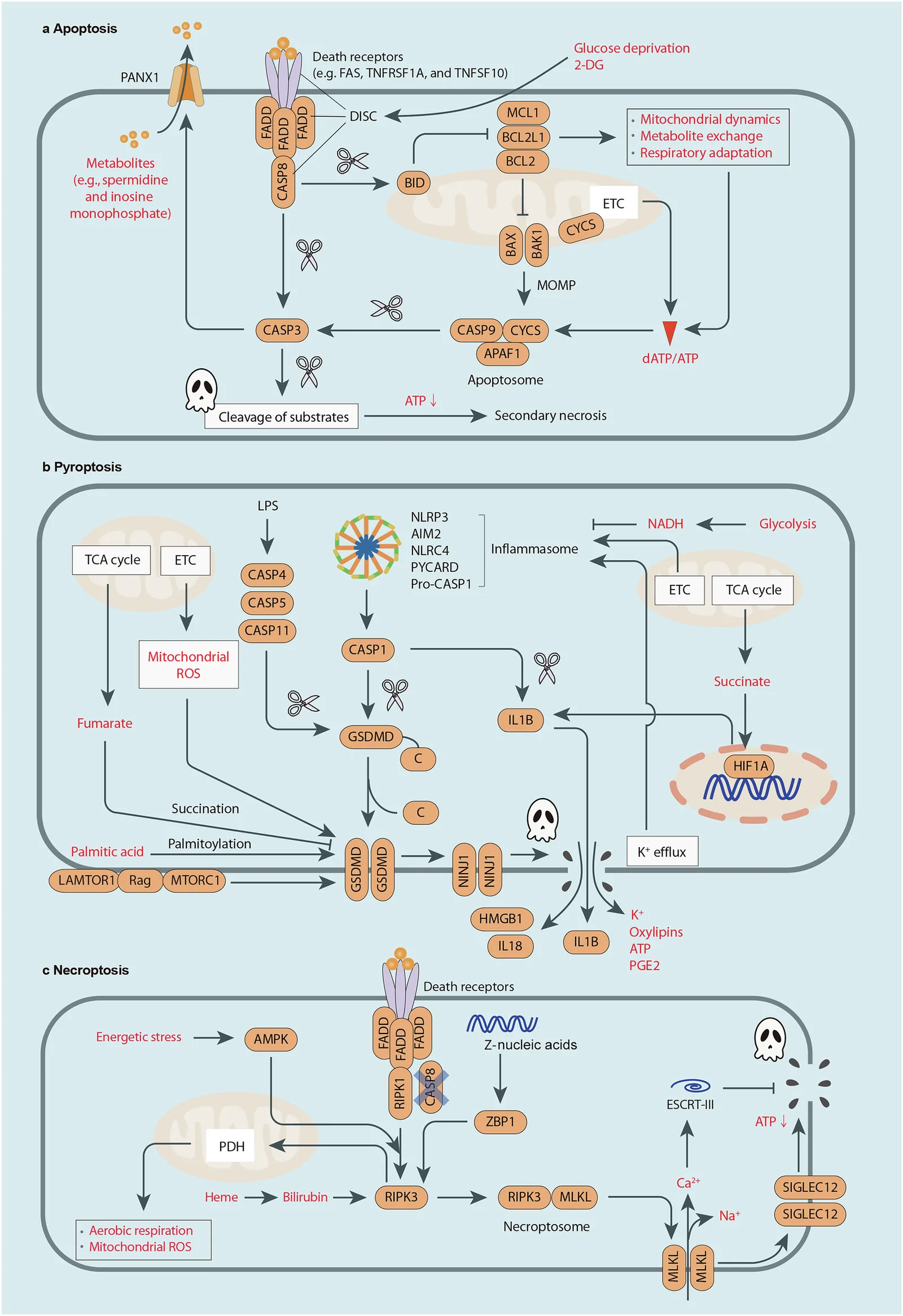
Metabolic regulation of apoptosis, pyroptosis, and necroptosis. **a** Apoptosis is a regulated form of cell death initiated through intrinsic mitochondrial or extrinsic death-receptor pathways. The intrinsic pathway is mediated by BCL2-associated X, apoptosis regulator (BAX)-BCL2 antagonist/killer 1 (BAK1)-dependent mitochondrial outer membrane permeabilization (MOMP), cytochrome c (CYCS) release, apoptosome assembly, and caspase activation. The extrinsic pathway is triggered by death receptors, including Fas cell surface death receptor (FAS), tumor necrosis factor (TNF) receptor superfamily member 1A (TNFRSF1A/TNFR1), and TNF superfamily member 10 (TNFSF10/TRAIL) receptors, leading to caspase-8 (CASP8) activation within the death-inducing signaling complex (DISC). Crosstalk between pathways occurs through CASP8-mediated cleavage of BH3-interacting domain death agonist (BID), linking death-receptor signaling to mitochondrial amplification. Apoptosis requires ATP/dATP for efficient caspase activation and apoptosome function. When ATP levels become critically depleted, apoptotic execution may fail, resulting in secondary necrosis. During apoptosis, caspase-dependent activation of pannexin 1 (PANX1) promotes the release of metabolites (e.g., spermidine and inosine monophosphate). **b** Pyroptosis is an inflammatory form of RCD driven by gasdermin (GSDM)-mediated membrane permeabilization. Canonical inflammasomes, including NLR family pyrin domain containing 3 (NLRP3), absent in melanoma 2 (AIM2), and NLR family CARD domain containing 4 (NLRC4), activate caspase-1 (CASP1), and cytosolic lipopolysaccharide activates caspase-4 (CASP4), caspase-5 (CASP5), or caspase-11 (CASP11) in non-canonical pathways. Gasdermin cleavage generates membrane pores that promote the release of interleukin-1 beta (IL1B), interleukin-18 (IL18), and damage-associated molecular patterns (DAMPs), and ninjurin 1 (NINJ1) mediates terminal plasma membrane rupture. Potassium (K^+^) efflux and metabolic reprogramming, including enhanced glycolysis, succinate accumulation, mitochondrial ROS production, and mechanistic target of rapamycin complex 1 (MTORC1) signaling, modulate inflammasome activation and gasdermin pore formation. Fumarate-dependent succination suppresses gasdermin D (GSDMD) activation and oligomerization, whereas GSDMD palmitoylation promotes pore activity. Inflammasome activation and gasdermin pore formation also promote the extracellular accumulation of oxylipins, ATP, and prostaglandin E2 (PGE2). **c** Necroptosis is a regulated necrotic cell death pathway driven by receptor-interacting serine/threonine kinase 3 (RIPK3)-dependent phosphorylation of MLKL, typically downstream of RIPK1 or RHIM-containing adapters such as Z-DNA binding protein 1 (ZBP1). Activated MLKL oligomerizes and translocates to cellular membranes, inducing permeabilization, ion influx, osmotic swelling, and membrane rupture. This pathway is often engaged when caspase-8 (CASP8) activity is inhibited. MLKL-driven Na^+^ and Ca^2+^ influx increases the energetic demand for ion pumping, accelerating ATP depletion and osmotic stress. A rapid increase in cytosolic Ca^2+^ also triggers recruitment of endosomal sorting complex required for transport III (ESCRT-III) to damaged membranes, where it promotes membrane repair. In parallel, RIPK3 can enhance metabolic activity and mitochondrial ROS production, further amplifying cellular injury. Mitochondrial metabolism and AMPK signaling modulate necroptotic progression under energetic stress, while excess unconjugated bilirubin can directly activate RIPK3 to trigger necroptosis. Additional components, including sialic acid binding Ig like lectin 12 (SIGLEC12), contribute to terminal membrane rupture downstream of MLKL activation.

Apoptosis is the most extensively studied form of RCD and can be initiated through intrinsic mitochondrial or extrinsic death-receptor pathways (Fig. 3a). The intrinsic pathway involves BAX (BCL2-associated X, apoptosis regulator)- and BAK1 (BCL2 antagonist/killer 1, also known as BAK)-mediated mitochondrial outer membrane permeabilization (MOMP), release of CYCS, apoptosome assembly, and subsequent activation of executioner caspases.^8,46,72,73^ The extrinsic pathway is triggered by so-called “death receptors”, including Fas cell surface death receptor (FAS), tumor necrosis factor (TNF) receptor superfamily member 1A (TNFRSF1A, also known as TNFR1), and TNF superfamily member 10 (TNFSF10, also known as TRAIL) receptors, which promote caspase-8 (CASP8) activation within death-inducing signaling complexes.^74,75^ Efficient death receptor-induced apoptosis also depends on CASP8-mediated cleavage of BH3-interacting domain death agonist (BID), which is a potent activator of BAX- and BAK1-dependent MOMP, linking the extrinsic and intrinsic pathways of apoptosis.^76^

The relationship between apoptosis and cellular metabolism has long been recognized. Early cell-free reconstitution experiments showed that CYCS-induced caspase activation requires deoxyadenosine triphosphate (dATP) or ATP, indicating that apoptosome assembly depends on the availability of energy-rich nucleotide triphosphates.^8,45^ Thus, apoptosis is not simply a passive consequence of catastrophic injury but proceeds most efficiently when at least part of the cellular metabolic machinery remains functional.

Mitochondria occupy a central position in the coupling between metabolism and apoptotic susceptibility. In addition to serving as reservoirs for CYCS and other apoptogenic factors, mitochondria integrate inputs related to nutrient availability, redox balance, and bioenergetic stress signals that together influence apoptotic priming. BCL2 family proteins participate in several aspects of this regulation. In addition to inhibiting MOMP, proteins such as BCL2-like 1 (BCL2L1, also known as BCL-XL) and MCL1 (MCL1 apoptosis regulator) influence mitochondrial dynamics, metabolite exchange, and respiratory adaptation.^73,77,78^ For example, interactions between BCL2L1 and voltage-dependent anion channels (VDACs) modulate mitochondrial metabolite flux and ATP/adenosine diphosphate (ADP) exchange,^79^ although the physiological significance of these interactions likely varies across cell types and stress conditions.

Cellular metabolic state can also influence apoptotic sensitivity. In several cancer models, inhibition of glycolysis by glucose deprivation or 2-deoxy-D-glucose (2-DG) enhances susceptibility to FAS- or TNFRSF1A-induced apoptosis by favoring the formation of the death-inducing signaling complex (DISC), resulting in the proteolytic processing and consequent enzymatic activation of proCASP8.^80^ Diverse configurations of mitochondrial metabolism can affect apoptotic susceptibility within tumors, enhancing the heterogeneity of this phenotype. In several therapy-resistant cancer models, drug-tolerant persister populations exhibit increased reliance on oxidative phosphorylation and fatty acid oxidation while remaining variably sensitive to BH3 mimetics.^81–83^ In acute myeloid leukemia, for example, the BCL2 inhibitor venetoclax initially induces apoptosis in susceptible cells, but resistant populations frequently display enhanced fatty acid oxidation and mitochondrial respiration.^82–84^ Pharmacological inhibition of these pathways can partially restore apoptotic sensitivity.^82–84^ However, the relationship between mitochondrial metabolic state and apoptotic priming appears to vary substantially across tumor types and treatment conditions.

ATP availability also affects the mode of cellular disassembly after apoptosis has been initiated. When mitochondrial injury becomes severe and ATP levels decline substantially, cells often fail to complete orderly apoptotic packaging and instead progress toward secondary necrosis.^20^ This transition is particularly evident in ischemic or inflammatory settings where bioenergetic recovery is limited.^85^ These observations demonstrate that apoptotic execution remains closely linked to cellular energetic status and that severe ATP depletion can shift cell death toward less organized forms of terminal disassembly.

The metabolic consequences of apoptosis extend beyond the dying cell itself. During apoptosis, caspase-mediated cleavage of pannexin 1 (PANX1) channels promotes the release of nucleotides and small metabolites before rupture of the plasma membrane.^86^ Metabolomic profiling of apoptotic Jurkat cells and primary thymocytes revealed the PANX1-dependent release of metabolites linked to nucleotide metabolism, glycolysis, and polyamine pathways, including spermidine and inosine monophosphate.^86^ In mouse models, exposure to these apoptotic metabolite mixtures altered transcriptional programs in neighboring myeloid cells and promoted anti-inflammatory or tissue repair-associated responses.^86^ Together, these results suggest that apoptotic cells can continue to participate in local metabolic communication even after commitment to death.

Sublethal apoptotic signaling may also reshape the state of the cell without causing its immediate elimination. In cancer models, incomplete or minority MOMP induced by chemotherapy or BH3 mimetics is associated with persistent mitochondrial stress, altered lipid metabolism, DNA damage signaling, and acquisition of drug-tolerant phenotypes.^49,87^ Surviving cells that experience sublethal apoptotic signaling may also exhibit increased susceptibility to ferroptosis, potentially because of the integrated stress response.^49^ However, the stability and physiological relevance of these transitional states are incompletely understood and may differ among experimental systems.

In summary, apoptosis can be considered a metabolically regulated process whose initiation, execution, and tissue-level consequences are all influenced by energetic state and mitochondrial function. Rather than functioning simply as an endpoint of mitochondrial injury, apoptotic signaling both responds to and reshapes cellular metabolism during stress adaptation and tissue remodeling.

### Pyroptosis

Pyroptosis is an inflammatory form of RCD driven by gasdermin-mediated membrane permeabilization (Fig. 3b). In canonical inflammasome signaling, microbial or sterile danger signals activate sensors such as NLR family pyrin domain containing 3 (NLRP3), absent in melanoma 2 (AIM2), or NLR family CARD domain containing 4 (NLRC4), leading to caspase-1 (CASP1) activation, gasdermin D (GSDMD) cleavage, and maturation of interleukin 1 beta (IL1B, also known as IL-1β) and interleukin 18 (IL18). In non-canonical pathways, cytosolic lipopolysaccharide (LPS) activates caspase-4 (CASP4) and caspase-5 (CASP5) in human cells or caspase-11 (CASP11) in mice, which likewise cleave GSDMD and induce pore formation in the plasma membrane.^10–13^ In addition to GSDMD, which appears to be the principal executor of pyroptosis, other members of the gasdermin family can mediate pyroptotic or pyroptosis-like membrane permeabilization in a context-dependent manner.^88–90^ Membrane permeabilization alone is not sufficient for complete cell lysis. Ninjurin 1 (NINJ1) functions as a mediator of terminal plasma membrane rupture downstream of membrane damage and contributes to cell lysis across multiple lytic death pathways as well as during mechanical injury.^91–97^

The metabolic dimension of pyroptosis is particularly evident in macrophages. LPS priming shifts macrophages toward glycolysis and remodels mitochondrial metabolism, thereby supporting inflammatory transcriptional programs and IL1B production.^40^ Interruption of the tricarboxylic acid (TCA) cycle further promotes succinate accumulation, stabilization of hypoxia-inducible factor 1 subunit alpha (HIF1A, also known as HIF1α), and enhanced inflammatory cytokine expression, linking metabolic rewiring directly to inflammasome-associated signaling.^40^ At the same time, inflammasome activation remains highly sensitive to metabolic imbalance. In LPS-primed macrophages, pharmacological disruption of glycolytic flux activates the NLRP3 inflammasome through an increased NAD^+^/NADH ratio and mitochondrial ROS generation, whereas restoration of NADH production suppresses pyroptotic signaling.^39^ Potassium efflux and activity of the mitochondrial electron transport chain also influence inflammasome competence across structurally diverse stimuli.^98–100^ These findings suggest that pyroptosis emerges not simply from inflammatory receptor activation but from a broader immunometabolic state in which nutrient utilization, redox balance, and ion homeostasis collectively shape inflammasome activation and gasdermin-mediated death.

Additional studies have demonstrated that metabolic regulation extends beyond inflammasome assembly and can directly influence membrane permeabilization. The LAMTOR1 (late endosomal/lysosomal adapter, MAPK and MTOR activator 1; also known as Ragulator)-Rag GTPase-MTORC1 (mechanistic target of rapamycin complex 1) pathway and mitochondrial ROS regulate GSDMD oligomerization and pore formation, linking nutrient perception and mitochondrial function to pyroptotic execution downstream of caspase activation.^39,100,101^ In parallel, fumarate-dependent succination suppresses GSDMD activation and oligomerization,^102^ whereas GSDMD palmitoylation promotes pore activity under inflammatory conditions.^103^ Pyroptosis is thus regulated not only at the level of inflammasome activation but also through metabolic and post-translational control of gasdermin pore formation and activity.

Pyroptosis also alters the extracellular metabolic environment after membrane permeabilization. In addition to the release of IL1B, IL18, and the alarmin high mobility group box 1 (HMGB1), inflammasome activation and gasdermin pore formation promote the extracellular accumulation of oxylipins, ATP, and other immunomodulatory metabolites.^104–108^ Some of these mediators, including prostaglandin E2 (PGE2), are linked to tissue repair and resolution programs in inflammatory settings, whereas persistent release of inflammatory metabolites can amplify local tissue injury.^107^ Taken together, pyroptosis exemplifies how immunometabolic remodeling involves the coordination of intracellular inflammasome activation and extracellular inflammatory communication, thus linking cellular metabolism to tissue-level immune responses during RCD.

### Necroptosis

Necroptosis is a regulated necrotic form of cell death mediated primarily by receptor-interacting serine/threonine kinase 3 (RIPK3)-dependent activation of MLKL, often downstream of RIPK1 or signaling proteins containing RIP homotypic interaction motifs (RHIMs, found in RIPK1 and RIPK3), such as Z-DNA binding protein 1 (ZBP1) (Fig. 3c).^41,109–113^ Once phosphorylated, MLKL undergoes conformational activation, oligomerization, and translocation to cellular membranes, particularly the plasma membrane, where it promotes membrane permeabilization, ion influx, osmotic swelling, and eventual rupture.^9,71,114^ This pathway is frequently engaged when CASP8 activity is inhibited or insufficient, especially during infection, inflammatory signaling, and tissue injury.^115,116^ Additional studies have identified sialic acid binding Ig like lectin 12 (SIGLEC12) as a component of the terminal necroptotic machinery downstream of MLKL, linking MLKL activation to plasma membrane rupture.^117^

Mechanistically, necroptosis involves a gradual destabilization of ionic and bioenergetic homeostasis rather than an instantaneous catastrophic event. MLKL-mediated membrane permeabilization promotes Na^+^ and Ca^2+^ influx, increasing the energetic demand required to maintain ion gradients and accelerating osmotic stress. Studies using inducible MLKL activation systems have demonstrated that plasma membrane translocation of MLKL or its N-terminal domain is sufficient to induce sodium influx, whereas reducing the level of extracellular sodium delays necroptotic death in cultured cells.^118^ These findings demonstrate that the MLKL-driven ionic imbalance increases bioenergetic stress and facilitates irreversible membrane rupture. Bioenergetic failure in necroptosis is primarily a consequence of escalating energetic demand imposed by loss of ion homeostasis rather than direct inhibition of ATP production.

The kinetics of necroptosis provide further support for this model. MLKL accumulation at the plasma membrane influences both the threshold and timing of membrane rupture. Live-cell imaging and biochemical analyses indicate that MLKL trafficking and membrane accumulation determine the rate of necroptotic execution, whereas limited MLKL activation can be temporarily restrained by membrane repair systems.^119^ Endosomal sorting complex required for transport III (ESCRT-III) is particularly relevant to this process. In TNF-induced necroptosis models, MLKL-mediated Ca^2+^ influx recruits ESCRT-III components to damaged membrane regions and promotes membrane shedding. Loss of ESCRT-III function accelerates membrane rupture, whereas intact ESCRT-III temporarily counteracts membrane damage and delays terminal cell lysis when MLKL activation remains submaximal.^120^ Thus, necroptotic commitment depends not only on MLKL activation itself but also on the balance between membrane damage, repair capacity, and the energetic cost of maintaining ion gradients. This dynamic balance demonstrates that necroptosis is not an instantaneous lytic event but a progressive metabolic process in which cellular repair mechanisms transiently counteract membrane injury before irreversible collapse occurs.

Necroptosis is best understood as a form of cell death characterized by progressive disruption of ionic balance and cellular energetics. Its defining metabolic lesion is not primary inhibition of ATP production but escalating failure of membrane and ion homeostasis that drives ATP consumption beyond the adaptive capacity of the cell. Cells can retain signaling competence during early stages of MLKL activation, and membrane repair pathways may transiently delay plasma membrane rupture. However, when ion influx, osmotic stress, ATP depletion, and membrane leakage exceed the capacity of cellular repair mechanisms, necroptosis progresses toward irreversible lytic death. This interpretation may help to explain why necroptosis is frequently implicated in ischemia-reperfusion injury and acute inflammatory tissue damage, where energetic stress, ionic imbalance, and inflammatory signaling often coexist.^121^ At the same time, disease models must be interpreted with caution because RIPK1, RIPK3, and MLKL also function independently of cell death, and pharmacological inhibition does not always distinguish necroptosis from broader inflammatory or metabolic effects.^122–124^

Mitochondria can influence necroptotic bioenergetics, although their contribution appears to vary across experimental systems. In some TNF-induced models, RIPK3 phosphorylates and activates the pyruvate dehydrogenase complex, thereby increasing aerobic respiration and mitochondrial ROS production during necroptosis.^125^ In these settings, enhanced mitochondrial metabolism feeds back onto necroptotic signaling and promotes cellular injury. However, mitochondrial respiration, mitochondrial permeability transition, and mitochondrial ROS are not universally required for necroptosis.^126^ These findings argue against a universal role for mitochondria as executors of necroptosis. Instead, mitochondrial metabolism appears to modulate the severity or irreversibility of necroptotic injury under selected conditions, particularly during pre-existing energetic or oxidative stress.

Energy-sensing pathways provide an additional point of interaction between necroptosis and cellular metabolism. AMP-activated protein kinase (AMPK), which is activated by an increase in the AMP:ATP and/or ADP:ATP ratio, modulates RIPK1-RIPK3 signaling under energetic stress and may transiently restrain necroptotic progression under certain conditions.^127,128^ Bilirubin toxicity provides a clinically relevant example of the link between metabolic dysfunction and necroptosis. Excess unconjugated bilirubin directly binds to and activates RIPK3, initiating the necroptotic signaling cascade and promoting MLKL-dependent membrane disruption.^129^ In neuronal and hepatic injury models, loss of RIPK3 attenuates bilirubin-induced cell death and tissue injury, supporting a functional role for necroptosis in bilirubin toxicity.^129^ Thus, nutrient-sensing mechanisms and metabolic stress can influence necroptotic susceptibility in a context-dependent manner.

### Ferroptosis

**Fig. 4:**
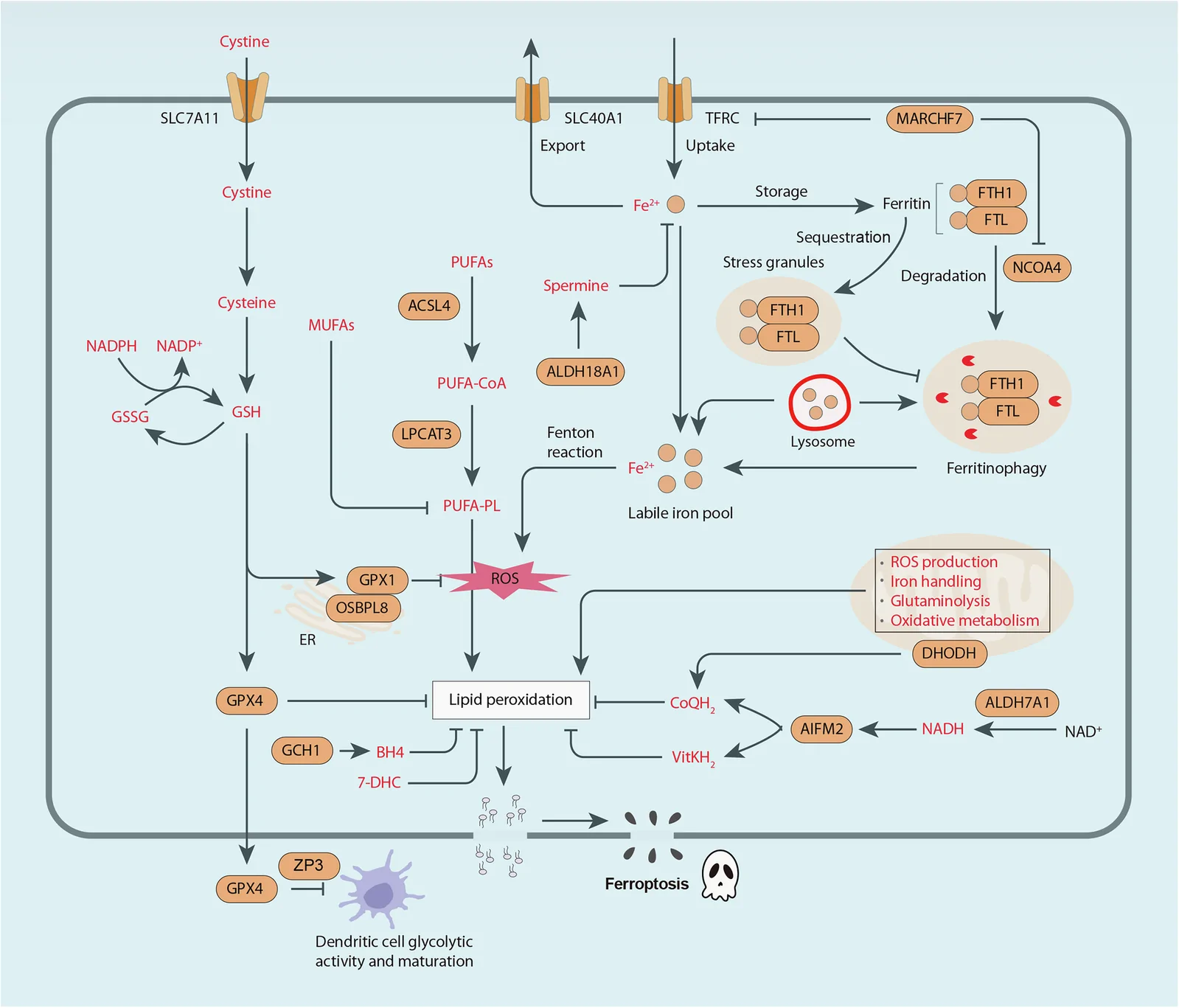
Metabolic regulation of ferroptosis. Ferroptosis is a non-apoptotic, iron-dependent form of cell death driven by lipid peroxidation. It is suppressed by iron chelators and lipophilic antioxidants and occurs when lipid peroxide generation exceeds antioxidant capacity. Central to this process is the glutathione (GSH)-glutathione peroxidase 4 (GPX4) axis, in which solute carrier family 7 member 11 (SLC7A11) promotes cystine uptake and GSH synthesis. Extracellular GPX4 released from ferroptotic cells suppresses glycolysis in dendritic cells through zona pellucida glycoprotein 3 (ZP3). Polyunsaturated fatty acid (PUFA)-containing phospholipids (PUFA-PLs), remodeled by acyl-CoA synthetase long chain family member 4 (ACSL4) and lysophosphatidylcholine acyltransferase 3 (LPCAT3), serve as major substrates for peroxidation, whereas enrichment of monounsaturated fatty acids (MUFAs) reduces susceptibility. NAD(P)H availability regulates GSH recycling and coenzyme Q (CoQ)-dependent antioxidant systems, linking ferroptosis sensitivity to glucose metabolism and reductive capacity. Additional GPX4-independent defenses include AIF family member 2 (AIFM2/FSP1)-mediated reduced CoQ (CoQH₂) or vitamin K (VitKH_2_) regeneration, dihydroorotate dehydrogenase (DHODH)-dependent mitochondrial CoQH₂ regeneration, 7-dehydrocholesterol (7-DHC), and the GTP cyclohydrolase 1 (GCH1)-tetrahydrobiopterin (BH4) axis. The glutathione peroxidase 1 (GPX1)-oxysterol binding protein like 8 (OSBPL8) pathway further contributes to ferroptosis suppression. Cellular iron homeostasis is tightly controlled by transferrin receptor 1 (TFRC)-mediated uptake and ferroportin (SLC40A1)-mediated export. Nuclear receptor coactivator 4 (NCOA4)-mediated ferritinophagy increases labile iron availability and promotes iron-dependent lipid peroxidation, and lysosomal iron release further amplifies redox-active iron pools. Stress granules suppress ferroptosis by sequestering ferritin (FTH1 and FTL), thereby limiting ferritinophagy. In addition, ALDH18A1-derived spermine functions as an endogenous iron chelator that limits ferroptotic damage, and membrane-associated Ring-CH-type finger 7 (MARCHF7) limits iron accumulation by promoting degradation of NCOA4 and restricting TFRC trafficking.

Ferroptosis was originally identified through chemical screening in *RAS*-mutant tumor cells treated with erastin,^130^ which induced a non-apoptotic form of RCD that could be suppressed by iron chelators and lipophilic antioxidants.^14^ Unlike apoptosis, ferroptosis does not involve chromatin condensation or caspase activation.^130^ Instead, ferroptosis develops when the production of phospholipid peroxides exceeds the capacity of cellular antioxidant systems (Fig. 4).^131^ A central component of this defense network is the GSH (glutathione)-GPX4 (glutathione peroxidase 4) axis. Erastin inhibits system xc^–^ through its solute carrier family 7 member 11 (SLC7A11, also known as xCT) subunit, limiting cystine uptake and reducing GSH synthesis, whereas direct inhibition (e.g., by RSL3) or genetic deletion of *GPX4* is sufficient to trigger ferroptosis even in the absence of upstream system xc^–^ inhibition.^26^ Ferroptosis shares biochemical features with oxytosis, an earlier form of neuronal cell death characterized by cystine deprivation, GSH depletion, and lipid peroxidation.^132^ In inducible *Gpx4*-deficient mouse models, spontaneous ferroptotic injury rapidly develops in the kidney, T cells, and nervous system, highlighting the essential role of GPX4 in limiting phospholipid peroxidation in vivo.^133–135^ Nevertheless, tissue-specific differences are evident. For example, conditional deletion of *Gpx4* in the pancreas does not impair normal pancreatic development but increases susceptibility to pancreatitis-associated injury, indicating that the physiological requirement for GPX4 varies across tissues.^136^ In addition to its intracellular antioxidant function, extracellular GPX4 may also influence the immunological consequences of ferroptosis.^137^

One of the most important conceptual advances in ferroptosis research is the recognition that membrane composition itself is a major determinant of death susceptibility. Polyunsaturated fatty acid (PUFA)-containing phospholipids are key substrates for peroxidation, and acyl-CoA synthetase long chain family member 4 (ACSL4)-dependent lipid remodeling promotes the incorporation of long-chain PUFAs into cellular membranes.^34–36^ Loss or pharmacological inhibition of ACSL4 reduces ferroptotic sensitivity in multiple cancer, tissue injury, and aging models by limiting the generation of peroxidation-prone phospholipid species.^138–140^ By contrast, enrichment of monounsaturated fatty acids (MUFAs) can reduce ferroptotic sensitivity, possibly by decreasing the abundance of peroxidation-susceptible phospholipids within cellular membranes.^141–144^ Other lipid species and membrane remodeling pathways also appear to influence ferroptotic sensitivity, although their relative contributions likely vary across cell types and metabolic contexts.^62,145,146^ Thus, ferroptosis depends not only on ROS accumulation but also on the availability of membrane lipids that are susceptible to peroxidation. This concept represents a major advance in our understanding of ferroptosis, shifting attention from oxidative stress alone toward membrane lipid remodeling as a fundamental determinant of ferroptotic susceptibility.

The relationship between mitochondria and ferroptosis is more complex than initially proposed. Mitochondria can contribute to ROS production, iron handling, and metabolic pathways linked to glutaminolysis and oxidative metabolism that influence ferroptotic sensitivity, particularly under cysteine deprivation or metabolic stress.^29^ However, ferroptosis can still occur in ρ^0^ cells depleted of mitochondrial DNA and in cells with compromised mitochondrial respiration, indicating that mitochondria are not universally required for ferroptotic execution and may instead function as context-dependent modulators of ferroptotic sensitivity.^14,147^ In practice, the contribution of mitochondrial metabolism to ferroptosis is influenced by cell type, nutrient availability, interactions with other metabolic organelles, and the mode of ferroptosis induction.^30,48,148–150^ These findings suggest that mitochondria should be viewed as context-dependent metabolic modulators of ferroptosis, whose contribution is determined by the metabolic state of individual cells and the nature of the initiating stress.

Redox metabolism nevertheless remains central to ferroptosis. NADPH availability influences GSH recycling, thiol reduction, and coenzyme Q (CoQ)-dependent antioxidant systems, linking ferroptosis sensitivity to glucose metabolism and broader reductive capacity.^32^ Under conditions of glucose limitation or impaired NADPH production, antioxidant defenses become less effective, rendering cells more vulnerable to the accumulation of lipid peroxides. Certain endogenous metabolites, including 7-dehydrocholesterol (7-DHC), can also inhibit phospholipid autoxidation and thereby suppress ferroptosis.^151,152^

Additional ferroptosis defense systems extend beyond the GSH-GPX4 axis. Among the best-characterized GPX4-independent suppressors of ferroptosis is AIF family member 2 (AIFM2, also known as FSP1), which limits ferroptotic injury independently of GSH by regenerating reduced CoQ (CoQH₂) at cellular membranes.^27,28^ Under some conditions, AIFM2-mediated vitamin K reduction also contributes to radical-trapping activity.^153,154^ The activity of AIFM2 is itself influenced by metabolic cofactor availability, as flavin adenine dinucleotide (FAD), generated from vitamin B2 (riboflavin), is required for AIFM2 stability and antioxidant function.^155,156^ Aldehyde dehydrogenase 7 family member A1 (ALDH7A1) also supports AIFM2-dependent ferroptosis defense by supplying membrane-associated NADH, detoxifying reactive aldehydes, and facilitating AIFM2 membrane localization.^157^ Additional membrane-protective functions of AIFM2, such as the AIFM2-mediated activation of ESCRT-III, have also been proposed.^158^ Recent studies suggest that GPX1 may participate in ferroptosis suppression under selected conditions. Through interaction with oxysterol-binding protein like 8 (OSBPL8), GPX1 can localize to the ER and reduce oxidized phosphatidic acid, thereby limiting ROS-driven lipid peroxidation.^159^ Whether this mechanism contributes broadly to ferroptosis control across tissues remains to be determined.

Dihydroorotate dehydrogenase (DHODH), a mitochondrial enzyme involved in pyrimidine biosynthesis, provides a parallel defense system by reducing CoQ within the inner mitochondrial membrane and suppressing mitochondrial lipid peroxidation, particularly in cells with low GPX4 activity.^50^ Inhibitor-based studies should be interpreted with caution owing to the reported off-target activities of some DHODH inhibitors toward AIFM2.^160^ Additional protection can be provided by the GTP cyclohydrolase 1 (GCH1)-tetrahydrobiopterin (BH4) pathway, which suppresses phospholipid peroxidation through mechanisms distinct from those of CoQ-dependent antioxidant systems.^161,162^ Thus, ferroptosis sensitivity reflects the balance between phospholipid oxidation and multiple, partially overlapping antioxidant defense systems rather than the failure of any one protective pathway.

Multiple metabolic systems also regulate iron accessibility. Among the best-characterized mechanisms are those that expand intracellular labile iron pools. Nuclear receptor coactivator 4 (NCOA4)-mediated ferritinophagy increases iron availability and promotes iron-dependent lipid peroxidation,^163,164^ and lysosomal iron release further contributes to the accumulation of redox-active iron.^165^ Ferroptosis therefore reflects the convergence of iron handling, membrane composition, and antioxidant metabolism into a self-reinforcing oxidative process. Conversely, endogenous iron-buffering mechanisms can restrict ferroptosis susceptibility. Aldehyde dehydrogenase 18 family member A1 (ALDH18A1) supports de novo spermine biosynthesis, and the resulting spermine acts as an endogenous iron chelator that restricts ferroptotic injury by limiting iron availability and suppressing lipid peroxidation.^166^ Consistent with a broader role for polyamine metabolism in ferroptosis control, polyamine depletion also exhibits synthetic lethality with GPX4 inhibition in leukemic cells.^167^ Similarly, the E3 ubiquitin ligase membrane-associated Ring-CH-type finger 7 (MARCHF7, also known as MARCH7) limits intracellular iron accumulation through degradation of NCOA4 and restriction of transferrin receptor (TFRC, also known as TFR1) translocation.^168^ Stress granules can also restrict ferroptosis by sequestering ferritin through G3BP stress granule assembly factor 1 (G3BP1)-dependent mechanisms, thereby limiting ferritinophagy and labile iron availability in glioblastoma stem cells.^169^ Thus, ferroptosis is determined not simply by total cellular iron content but by dynamic regulation of the labile iron pool, emphasizing that iron accessibility rather than iron abundance is the critical metabolic determinant of ferroptotic susceptibility.

Ferroptosis blurs the distinction between metabolic dysfunction and cell death execution mechanisms. The metabolic processes that generate oxidizable phospholipids, regulate iron availability, and maintain antioxidant defenses also determine susceptibility to ferroptotic injury. Ferroptosis may also extend beyond the initially affected cell, as oxidized lipid species can propagate oxidative injury and increase the ferroptotic susceptibility of neighboring cells.^170,171^ Recent work has implicated lysosomal membrane damage in the propagation of ferroptosis, suggesting that mechanisms of ferroptosis execution may differ between isolated cellular responses and tissue-level propagation.^172^ Whether the cell death that occurs during such propagation fully conforms to the molecular definition of ferroptosis remains to be determined.^173^

Ferroptosis thus demonstrates how metabolic regulation and cell death execution are functionally inseparable. Ferroptosis emerges from the integration of membrane lipid remodeling, iron availability, antioxidant capacity, mitochondrial metabolism, and inter-organelle communication, all of which collectively determine whether cells cross the threshold for irreversible lipid peroxidation and membrane damage.

## Independently validated forms of RCD

### Lysosome-dependent cell death

**Fig. 5:**
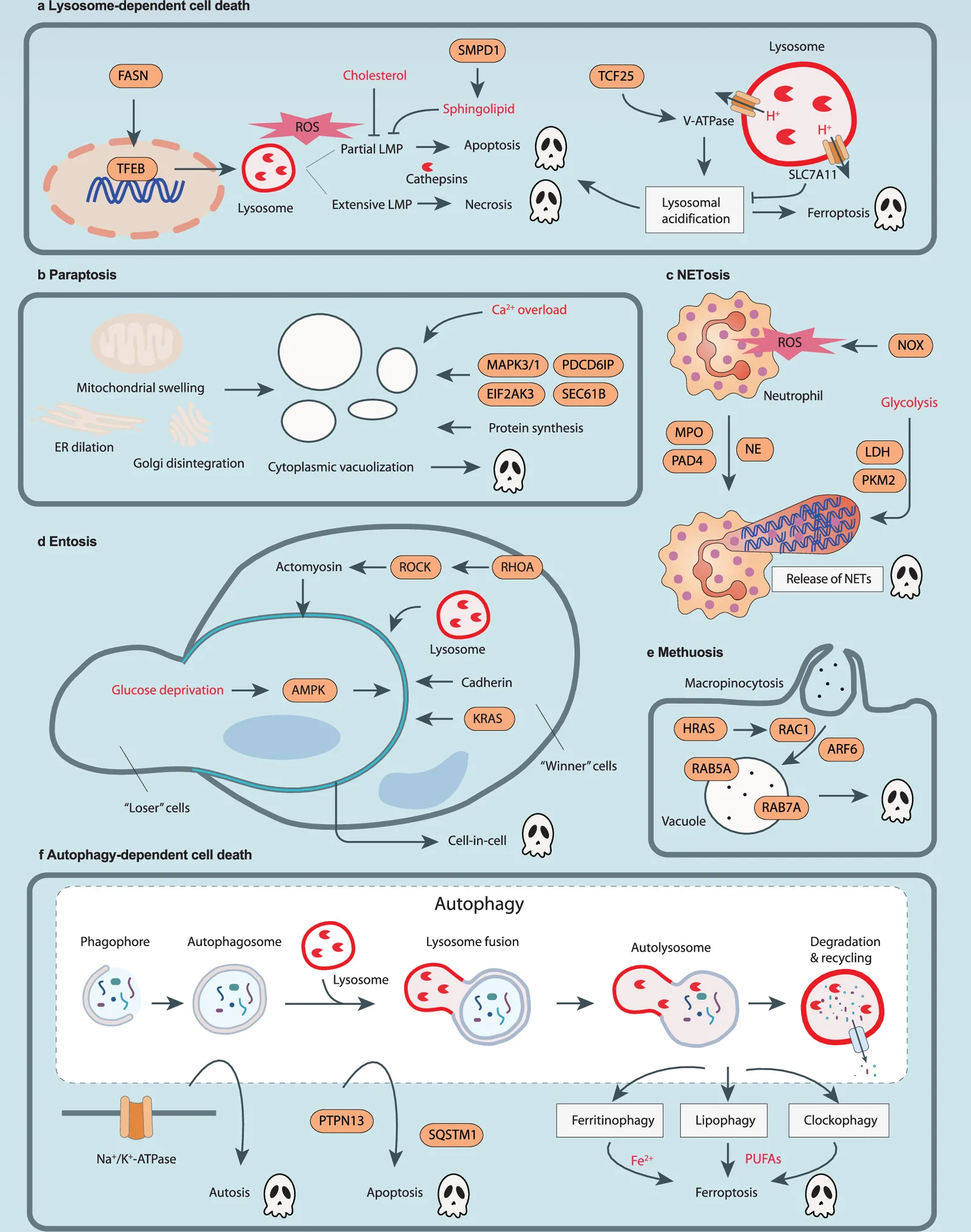
Metabolic regulation of lysosome-dependent cell death, paraptosis, NETosis, entosis, methuosis, and autophagy-dependent cell death. **a** Lysosome-dependent cell death is initiated by lysosomal membrane permeabilization (LMP), resulting in the release of cathepsins and other lysosomal contents into the cytosol. Depending on the extent of lysosomal damage, LMP can promote apoptotic, necrotic, or ferroptotic features. Sphingolipid imbalance caused by inhibition of sphingomyelin phosphodiesterase 1 (SMPD1), transcription factor EB (TFEB)-associated lysosomal stress induced by inhibition of fatty acid synthase (FASN), and lysosomal fragility mediated by TCF25 ribosome quality control complex subunit (TCF25) during glucose deprivation can all trigger lysosome-associated cell death. Lysosomal solute carrier family 7 member 11 (SLC7A11) further links lysosomal function to ferroptosis susceptibility through regulation of lysosomal pH and amino acid transport. **b** Paraptosis is a non-apoptotic cell death process characterized by extensive cytoplasmic vacuolization derived primarily from ER dilation and, in some contexts, mitochondrial swelling. ER stress, calcium dysregulation, mitochondrial dysfunction, and sustained activation of mitogen-activated protein kinase 3/1 (MAPK3/1, also known as ERK1/2) are commonly observed. Additional regulators include programmed cell death 6-interacting protein (PDCD6IP/ALIX), eukaryotic translation initiation factor 2 alpha kinase 3 (EIF2AK3/PERK), and SEC61 translocon subunit beta (SEC61B), and inhibition of protein synthesis often suppresses paraptotic vacuolization. Golgi disintegration may also occur during paraptosis progression. **c** NETosis is an inflammatory neutrophil response characterized by the release of neutrophil extracellular traps (NETs) composed of chromatin, histones, and granule proteins. NADPH oxidase (NOX)-dependent ROS production, peptidylarginine deiminase 4 (PAD4), myeloperoxidase (MPO), and neutrophil elastase (NE) contribute to NET formation. Neutrophils rely predominantly on glycolytic metabolism, and glycolytic rewiring, lactate dehydrogenase (LDH) activity, and pyruvate kinase M2 (PKM2) activation are closely linked to NET release. **d** Entosis is a cell-in-cell death process in which a viable cell actively invades a neighboring cell and is subsequently degraded within the host cell through lysosome-dependent mechanisms. Unlike phagocytosis, internalization is driven by the invading cell through Ras homolog family member A (RHOA)-Rho-associated coiled-coil containing protein kinase (ROCK)-dependent actomyosin contractility and cadherin-mediated adhesion. Metabolic stress induced by glucose deprivation activates AMPK signaling and promotes the emergence of “loser” cells that are preferentially engulfed by neighboring “winner” cells. Oncogenic KRAS signaling contributes to winner-loser cell competition. **e** Methuosis is a non-apoptotic cell death pathway driven by dysregulated macropinocytosis and excessive accumulation of macropinosome-derived vacuoles. Sustained activation of Rac family small GTPase 1 (RAC1), impaired ARF GTPase 6 (ARF6)-mediated membrane recycling, and defective endosomal trafficking promote vacuole formation; altered RAB5A- and RAB7A-dependent vesicular maturation further exacerbates intracellular membrane dysregulation, ultimately leading to cell death. **f** Autophagy-dependent cell death requires the autophagic machinery. Excessive or dysregulated autophagy can directly promote cell death, as exemplified by autosis, a Na^+^/K^+^-ATPase-associated death pathway induced by sustained autophagic activation. Selective autophagy can also facilitate other forms of RCD, including apoptosis through sequestosome 1 (SQSTM1/p62)-dependent degradation of protein tyrosine phosphatase non-receptor type 13 (PTPN13) and ferroptosis through ferritinophagy, lipophagy, and clockophagy.

Lysosome-dependent cell death is characterized by lysosomal membrane permeabilization (LMP) and leakage of cathepsins and other lysosomal contents into the cytosol (Fig. 5a).^53^ This process is frequently studied using lysosomotropic agents that accumulate within acidic lysosomal compartments and promote LMP, including compounds such as siramesine, L-leucyl-L-leucine methyl ester (LLOMe), and other cationic amphiphilic drugs.^174–176^ In cancer cells, lysosomal destabilization can also be triggered by inhibition of sphingomyelin phosphodiesterase 1 (SMPD1, also known as ASM), which leads to sphingolipid imbalance, LMP, and lysosome-dependent cell death.^177^ Depending on the extent of lysosomal damage and the cellular context, LMP can promote apoptotic, necrotic, or ferroptotic features rather than always defining a separate death pathway. Cells normally counter limited lysosomal injury through membrane repair and lysophagy-mediated quality control.^178–185^ However, once damage exceeds repair capacity, lysosomal rupture propagates proteolytic stress, redox imbalance, and membrane injury across the cell.^186^

Lysosomes function both as degradative compartments and as metabolic signaling organelles that regulate nutrient recycling, lipid turnover, and autophagic flux. Partial LMP can release cathepsins that amplify mitochondrial permeabilization and apoptotic signaling,^187,188^ whereas extensive lysosomal rupture causes broader proteolytic damage and necrotic collapse.^189^ The outcome of lysosomal injury, therefore, depends not only on cathepsin release but also on membrane composition, degradative capacity, metabolic state, and the ability of cells to repair damaged lysosomes. For example, cholesterol accumulation stabilizes lysosomal membranes and reduces susceptibility to oxidative injury, whereas cholesterol depletion has the opposite effect.^190^ Lipid metabolic rewiring can likewise influence lysosomal fragility, as inhibition of fatty acid synthase (FASN) activates transcription factor EB (TFEB)-associated lysosomal stress programs and promotes lysosome-associated death in leukemia models.^191^

Lysosomal adaptation itself can become maladaptive during persistent nutrient stress. Under glucose starvation, TCF25 ribosome quality control complex subunit (TCF25)-dependent activation of vacuolar H^+^-ATPase (V-ATPase) enhances lysosomal acidification, autophagy, and ATP maintenance during early adaptation, but prolonged activation ultimately increases lysosomal fragility and promotes cell death.^192^ Lysosomal dysfunction also intersects with ferroptosis.^150,165,193–196^ In addition, lysosomal SLC7A11 regulates lysosomal pH through proton-coupled cystine and glutamate flux, linking lysosomal function to ferroptosis susceptibility and neurodegenerative pathology.^197^

A key feature of lysosome-dependent cell death is the loss of degradative compartmentalization. Lysosomes normally sequester hydrolytic enzymes, damaged macromolecules, and metabolic waste within a specialized acidic environment. Once this spatial organization is disrupted, proteolytic stress and metabolic dysfunction spread throughout the cell and amplify injury in other organelles. Accordingly, lysosomal damage is increasingly recognized not as a single execution mechanism but as a disruption of intracellular compartmental organization that can contribute to multiple forms of RCD.

### Paraptosis

Paraptosis is characterized by extensive cytoplasmic vacuolization, typically associated with dilation of the ER and, in some settings, mitochondrial swelling. It was originally described in insulin-like growth factor I receptor-overexpressing cells undergoing receptor hyperactivation.^59^ Paraptosis typically occurs without caspase activation, chromatin condensation, or apoptotic body formation.

Similar vacuolization-associated death phenotypes have subsequently been reported in cancer and non-cancer cells exposed to proteasome inhibitors, natural products, viral infection, and other stimuli that disrupt proteostasis, induce ER stress, or alter intracellular metabolic homeostasis, including heme accumulation and intracellular acidification.^198–204^ A common feature of these models is the self-reinforcing loss of organelle homeostasis. ER stress, calcium dysregulation, mitochondrial dysfunction, and sustained mitogen-activated protein kinase 3/1 (MAPK3/1, also known as ERK1/2) activation is frequently observed, whereas inhibition of protein synthesis often suppresses vacuole formation and cell death (Fig. 5b).^201,205–207^ Additional regulators, including programmed cell death 6-interacting protein (PDCD6IP, also known as ALIX), eukaryotic translation initiation factor 2 alpha kinase 3 (EIF2AK3, also known as PERK), and SEC61 translocon subunit beta (SEC61B), are implicated in specific experimental settings.^207,208^

Despite these recurring features, the molecular basis of paraptosis is less well defined. No dedicated execution machinery has been identified, and many proposed mechanisms overlap with broader responses to proteotoxic stress, unfolded protein response signaling, and organelle dysfunction. Golgi disintegration has also been reported during paraptotic progression, although its functional significance remains unclear.^209^ Thus, paraptosis may represent a vacuolization-associated organelle stress response that can emerge from diverse forms of metabolic and proteostatic disruption rather than a distinct and uniformly regulated death pathway.

### NETosis

NETosis refers to inflammatory neutrophil responses characterized by the release of neutrophil extracellular traps (NETs) composed of chromatin, histones, and granule proteins. Although some types of NET formation can occur independently of terminal cell lysis, NET release is included here because it is frequently coupled to metabolically regulated membrane rupture (Fig. 5c).^210,211^ These structures contribute to antimicrobial defense but can also promote thrombosis, autoimmunity, and tissue injury when excessive or unresolved.^211–215^ Mechanistically, NETosis can proceed through both NADPH oxidase-dependent and -independent pathways.^216^ NET formation influences tumor progression and therapy resistance, in part through remodeling of the tumor immune microenvironment.^217^

NETosis is closely linked to glycolytic metabolism and ROS production.^218,219^ In phorbol 12-myristate 13-acetate (PMA)-induced NETosis, NADPH oxidase-derived ROS promote activation of peptidylarginine deiminase 4 (PAD4), histone citrullination, and chromatin decondensation, while neutrophil elastase and myeloperoxidase further facilitate NET release. However, the contribution of neutrophil serine proteases to NET formation appears to vary across experimental settings, and catalytic protease activity may not be universally required for NETosis.^220^ Because neutrophils possess limited mitochondrial respiratory capacity and rely heavily on glycolytic metabolism, the oxidative burst and ATP production remain tightly interconnected during NET release. Additional studies have shown that both NADPH oxidase (NOX)-dependent and NOX-independent NETosis are associated with increased glycolytic flux, lactate production, and pyruvate kinase M2 (PKM2) activation, whereas pharmacological inhibition of lactate dehydrogenase (LDH) suppresses NET formation in vitro and in vivo.^221–224^ These studies indicate that lactate metabolism contributes directly to NET formation rather than representing a secondary consequence of neutrophil activation.

NETosis is also shaped by intercellular metabolic signaling within inflammatory microenvironments. Platelet-derived oxidized phospholipids promote PAD4-dependent NET formation through platelet-neutrophil crosstalk, linking dysregulated lipid metabolism and inflammatory signaling to immunothrombosis and vascular injury.^225^ Damage-associated molecular patterns (DAMPs) such as HMGB1 and S100 family proteins can further amplify NET formation and inflammatory propagation.^226,227^

NETosis is increasingly recognized as a process shaped by broader metabolic programs rather than by ROS production alone. Although ROS remains central in many models, glycolytic rewiring, lactate metabolism, and inflammatory metabolic stress also influence the ability of neutrophils to sustain NET release, particularly within hypoxic or inflamed tissues.^228,229^ NETosis therefore reveals how the functions of inflammatory effectors and lytic cell injury can become metabolically inseparable in highly specialized immune cells.

### Entotic cell death

Entotic cell death (entosis) is a cell-in-cell process in which a viable cell actively invades a neighboring cell and is subsequently eliminated through lysosome-dependent degradation within the host cell (Fig. 5d).^230^ Unlike phagocytosis, the internalized cell actively contributes to its own uptake through RHOA (Ras homolog family member A)-ROCK (Rho-associated coiled-coil containing protein kinase)-dependent actomyosin contractility and cadherin-mediated cell-cell adhesion.^230–233^ Originally described in matrix-detached epithelial cells, entosis is now recognized as a broader mechanism of cell competition in which relative cellular fitness influences cell survival and elimination.^234,235^

The relationship between entosis and metabolism is most apparent under conditions of nutrient limitation. Glucose deprivation activates AMPK signaling and promotes the emergence of metabolically stressed “loser” cells that are preferentially internalized by neighboring “winner” cells.^236^ Degradation of internalized cells provides nutrients to host cells and thereby supports survival under starvation conditions. Consistent with this model, oncogenic *KRAS* signaling can confer winner-cell behavior, whereas cadherin-dependent polarization of RHOA signaling and actomyosin contractility contributes to the establishment of winner-loser asymmetry.^234,235^ These studies suggest that entosis functions as a tissue-level adaptation to metabolic stress that links nutrient availability, cellular fitness, and selective cell elimination.

The biological consequences of entosis are highly context-dependent. On one hand, entosis may promote tumor progression by providing nutrients to surviving cells and by inducing cytokinesis failure, multinucleation, and aneuploidy, thereby facilitating clonal evolution and genome instability.^237^ Through interference with normal cell division, entosis can also trigger mitotic catastrophe-like abnormalities, providing a non-genetic route to chromosomal instability in cancer. Cell-in-cell structures have also been associated with aggressive disease behavior and poor prognosis in several malignancies, although their prognostic significance may vary among tumor types.^238^ On the other hand, entosis and related cell-in-cell elimination processes may exert tumor-suppressive effects by eliminating less-fit or potentially harmful cells.^239^

Although most extensively studied in cancer, entosis-like processes also participate in normal physiology and disease. During embryo implantation, trophoblast cells remove uterine luminal epithelial cells through an entosis-like mechanism that facilitates tissue remodeling and embryo-maternal interaction.^240^ Conversely, excessive cell-in-cell formation is implicated in developmental pathology. In PALS1 (protein associated with LIN7 1, MAGUK P55 family member)-deficient mouse models, abundant cell-in-cell structures accompany mitotic abnormalities, cell death, and cortical hypoplasia, suggesting that entosis-associated mechanisms may contribute to the pathogenesis of microcephaly.^241^ Taken together, these studies indicate that entosis is not restricted to cancer but participates more broadly in cell competition, tissue remodeling, and cellular quality control in diverse physiological and pathological settings.

Related heterotypic cell-in-cell interactions can result in distinct outcomes. For example, internalized cytotoxic lymphocytes may undergo granzyme B (GZMB)-dependent apoptotic death (emperitosis), a process proposed to contribute to tumor immune evasion.^242^ Because its execution relies on canonical apoptotic machinery, emperitosis is generally regarded as a specialized outcome of heterotypic cell-in-cell interactions rather than a distinct death modality.

### Methuosis

Methuosis is a non-apoptotic cell death process driven by dysregulated macropinocytosis and vacuole accumulation.^243^ It was originally identified in glioblastoma cells expressing constitutively active *HRAS*, where excessive macropinocytic uptake generates large phase-lucent vacuoles derived from macropinosomes and late endosomal compartments (Fig. 5e).^244^ These vacuoles fail to mature or recycle properly, progressively accumulate within the cytoplasm, and eventually disrupt intracellular organization and cellular function.

Mechanistically, methuosis is associated with sustained activation of Rac family small GTPase 1 (RAC1), impaired ARF GTPase 6 (ARF6)-dependent membrane recycling, and defects in endosomal trafficking.^244–248^ Altered RAB5A- and RAB7A-regulated vesicular maturation further contributes to vacuole accumulation in some models.^245^ Similar phenotypes have been reproduced using small molecules that stimulate macropinocytosis or disrupt endolysosomal trafficking.^249–251^

Methuosis reveals how a normally adaptive nutrient acquisition pathway can become self-destructive when vesicular trafficking is no longer properly coordinated. In *RAS*-driven tumors, macropinocytosis can support growth by providing extracellular proteins as metabolic substrates. However, excessive vesicular influx coupled with defective recycling converts this adaptive process into a source of cellular stress. Sustained vacuole accumulation disrupts membrane turnover, intracellular compartment organization, and energy balance, eventually leading to non-apoptotic cell death.^252^

Although methuosis is considered a distinct vacuolization-associated phenotype, its molecular boundaries are incompletely defined and overlap with broader defects in endosomal trafficking and membrane dynamics.^253^

### Autophagy-dependent cell death

Autophagy is a conserved stress-response pathway that recycles nutrients, removes damaged organelles, and supports cellular adaptation during stress. Under nutrient limitation, hypoxia, or mitochondrial injury, autophagic flux supplies substrates that help to sustain cellular function.^254^ Nonetheless, persistent or excessive autophagy can contribute to cell death.

A major challenge in the field has been distinguishing “autophagic cell death” from true “autophagy-dependent cell death”.^255^ Historically, autophagic cell death referred primarily to cell death that was accompanied by prominent autophagic structures. However, in many settings, autophagy appears to be associated with, rather than required for, the death process. By contrast, autophagy-dependent cell death is defined by experimental evidence showing that inhibition of core autophagy machinery suppresses cell death itself rather than merely altering accompanying phenotypes. One example is *Drosophila* salivary gland regression, in which the disruption of autophagy delays developmental tissue elimination despite ongoing apoptotic signaling, indicating that autophagic degradation contributes directly to cell destruction.^256^ Another example is autosis, a Na^+^/K^+^-ATPase-associated form of autophagy-dependent death triggered by excessive autophagic activation during starvation, hypoxia, or ischemic stress.^257–259^

Selective autophagy can also influence cell fate and death-pathway choice by determining which intracellular components are targeted for degradation (Fig. 5f). For example, sequestosome 1 (SQSTM1, also known as p62)-dependent autophagic degradation of the anti-apoptotic regulator protein tyrosine phosphatase non-receptor type 13 (PTPN13, also known as FAP1) enhances FAS signaling and facilitates CASP8-dependent apoptosis.^260^ Other forms of selective autophagy, including ferritinophagy,^163,164^ lipophagy,^64,261^ and clockophagy,^262,263^ can regulate iron homeostasis, lipid metabolism, and circadian signaling through the degradation of specific cargoes. In these examples, autophagy does not simply accompany ferroptosis but can actively contribute to its execution, a process often referred to as autophagy-dependent ferroptosis.^55^

In this context, autophagy-dependent death marks the point at which adaptive recycling becomes self-destructive. Early autophagy supports cellular function through nutrient recovery and organelle quality control, whereas persistent activation can progressively undermine metabolic adaptation and contribute to cell death. The outcome of autophagy is influenced both by the extent of autophagic activity and by the identity of the degradation cargo and the metabolic conditions in which autophagic flux occurs.

### Parthanatos

**Fig. 6:**
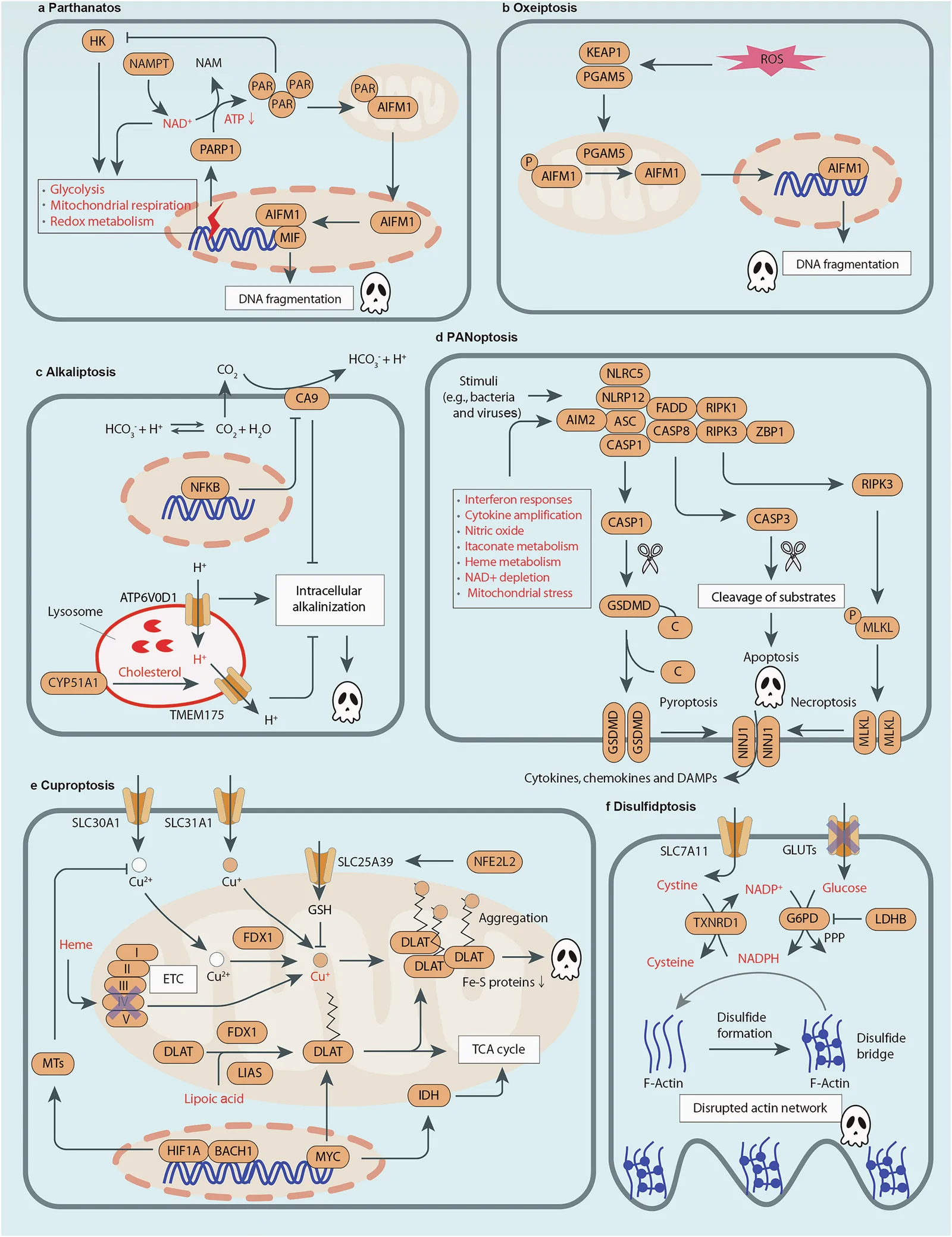
Metabolic regulation of parthanatos, oxeiptosis, alkaliptosis, PANoptosis, cuproptosis, and disulfidptosis. **a** Parthanatos is a PARP1-dependent form of cell death induced by excessive DNA damage and PARP1 hyperactivation. PARP1 overactivation leads to accumulation of poly(ADP-ribose) (PAR) polymers, triggering mitochondrial release of apoptosis-inducing factor mitochondria-associated 1 (AIFM1) and its nuclear translocation with macrophage migration inhibitory factor (MIF), resulting in large-scale DNA fragmentation. PARP1 consumes NAD^+^ during DNA repair, and sustained activation leads to NAD^+^ depletion and collapse of glycolytic, mitochondrial, and redox metabolism. PAR polymers further inhibit glycolysis by suppressing hexokinase (HK) activity, thus amplifying energetic failure. NAD⁺ regeneration via nicotinamide phosphoribosyltransferase (NAMPT) is essential for maintaining PARP1 activity and cellular metabolic homeostasis. **b** Oxeiptosis is an ROS-induced, caspase-independent form of RCD mediated by the Kelch-like ECH-associated protein 1 (KEAP1)-PGAM family member 5, mitochondrial serine/threonine protein phosphatase (PGAM5)-AIFM1 signaling axis. Elevated ROS levels disrupt KEAP1-PGAM5 interactions, enabling PGAM5-dependent activation of AIF family member 1 (AIFM1)-associated DNA fragmentation. **c** Alkaliptosis is a pH-dependent form of RCD characterized by intracellular alkalinization. Mechanistically, nuclear factor kappa-light-chain-enhancer of activated B cells (NFKB)-mediated repression of carbonic anhydrase 9 (CA9) disrupts proton buffering and impairs intracellular pH homeostasis. Lysosomal ATPase H^+^-transporting V0 subunit D1 (ATP6V0D1)-dependent lysosomal acidification enhances sensitivity to alkaliptosis. In addition, cytochrome P450 family 51 subfamily A member 1 (CYP51A1)-mediated cholesterol metabolism limits lysosomal cholesterol accumulation and promotes transmembrane protein 175 (TMEM175)-dependent lysosomal proton flux, thereby suppressing alkaliptotic cell death. **d** PANoptosis is an inflammatory lytic cell death pathway in which pyroptotic, apoptotic, and necroptotic signaling modules become functionally integrated through PANoptosome assembly. Distinct PANoptosome complexes organized by sensors such as Z-DNA binding protein 1 (ZBP1), absent in melanoma 2 (AIM2), receptor-interacting serine/threonine kinase 1 (RIPK1), NLR family CARD domain containing 5 (NLRC5), and NLR family pyrin domain containing 12 (NLRP12) coordinate the activation of caspases (CASPs), gasdermins (GSDMs), mixed lineage kinase domain like pseudokinase (MLKL), and ninjurin 1 (NINJ1). Cytokine signaling, interferon responses, nitric oxide-associated metabolic disruption, mitochondrial stress, and NAD^+^ depletion can lower the threshold for coordinated activation of multiple death programs. **e** Cuproptosis is a copper-dependent form of cell death closely linked to mitochondrial TCA cycle activity. Copper binds to lipoylated components of the TCA cycle, including dihydrolipoamide S-acetyltransferase (DLAT), leading to the aggregation of lipoylated proteins and the loss of iron-sulfur cluster (Fe-S) proteins. Ferredoxin 1 (FDX1) regulates protein lipoylation via lipoic acid synthetase (LIAS), thereby coupling cuproptosis to mitochondrial metabolic homeostasis. NFE2-like BZIP transcription factor 2 (NFE2L2/NRF2)-dependent expression of solute carrier family 25 member 39 (SLC25A39) promotes mitochondrial glutathione (GSH) uptake and suppresses cuproptotic stress by buffering oxidative damage and labile copper levels. Increased metallothionein (MT) expression downstream of hypoxia-inducible factor 1 subunit alpha (HIF1A) activation or BACH transcriptional regulator 1 (BACH1) loss reduces copper toxicity, whereas MYC proto-oncogene, BHLH transcription factor (MYC)-driven upregulation of DLAT and isocitrate dehydrogenase (NADP^+^) 1 (IDH1) enhances susceptibility to cuproptosis. In addition, low-dose heme can destabilize mitochondrial complex IV, promote copper release, and induce cuproptosis-like cell death. **f** Disulfidptosis is a disulfide stress-driven cell death pathway that occurs in solute carrier family 7 member 11 (SLC7A11)-high cells under glucose deprivation. Continued cystine uptake under conditions of impaired NADPH production leads to intracellular disulfide accumulation instead of reduction to cysteine. This redox imbalance results in aberrant disulfide bonding in cytoskeletal proteins, leading to F-actin collapse and rapid cell death. Inhibition of thioredoxin reductase 1 (TXNRD1) or disruption of the lactate dehydrogenase B (LDHB)-glucose-6-phosphate dehydrogenase (G6PD) axis further enhances disulfide stress by limiting cellular reducing capacity.

Parthanatos is initiated by severe DNA damage and excessive activation of PARP1 (Fig. 6a).^264^ Unlike apoptosis, parthanatos does not depend on caspase activation. Instead, PARP1 hyperactivation promotes the accumulation of poly(ADP-ribose) (PAR) polymers, which contribute to the release of apoptosis-inducing factor mitochondria-associated 1 (AIFM1, also known as AIF) from mitochondria and its subsequent nuclear translocation, followed by large-scale DNA fragmentation and cell death.^265,266^ Macrophage migration inhibitory factor (MIF) functions downstream of AIFM1 as a nuclease required for large-scale DNA fragmentation during parthanatos.^267^

Parthanatos is distinguished by the extent to which bioenergetic collapse arises from excessive NAD^+^ consumption during the DNA damage response. PARP1 consumes NAD⁺ during DNA repair reactions, and its sustained activation can deplete intracellular NAD^+^ pools. Because NAD^+^ is an essential cofactor for glycolysis, mitochondrial respiration, and redox metabolism, excessive PARP1 activity disrupts multiple pathways required for bioenergetic homeostasis.^265^ Live-cell metabolic imaging studies have shown that PARP1 activation rapidly alters cellular NADH dynamics after DNA damage, consistent with early disturbance of NAD^+^-linked metabolic regulation.^268^

The metabolic consequences of PARP1 hyperactivation extend beyond NAD^+^ depletion. In neuronal and astrocytic models exposed to oxidative or genotoxic stress, PARP1 activation is associated with impaired glycolytic flux, ATP decline, and escalating energetic failure.^265,269^ Restoration of NAD^+^ availability or pharmacological inhibition of PARP1 partially preserves glycolytic activity, attenuates energetic collapse, and reduces cell death in several experimental systems.^265^ In some models, PAR polymers generated after PARP1 activation inhibit hexokinase activity and suppress the first committed step of glycolysis, suggesting that parthanatos involves both the depletion of metabolic cofactors and the direct disruption of glycolytic control.^269,270^ PAR polymers can also amplify proteotoxic stress. In neuronal models of Parkinson disease, PAR promotes the formation of toxic α-synuclein aggregates, thereby contributing to parthanatos-associated neurodegeneration.^271^

NAD^+^ salvage pathways influence the threshold between metabolic adaptation and energetic collapse. Nicotinamide phosphoribosyltransferase (NAMPT)-dependent NAD^+^ regeneration maintains the substrate pool required for PARP1 activity, but persistent NAD^+^ salvage can also sustain PARP1-driven NAD^+^ turnover under chronic oxidative stress.^272,273^ Under conditions of mild H_2_O_2_ exposure, NAMPT-dependent NAD^+^ salvage enables stressed cells to undergo apoptosis, and its inhibition favors the depletion of NAD^+^ and ATP, resulting in necrotic collapse. Thus, NAD^+^ metabolism helps to shape death-pathway selection in addition to functioning as a protective mechanism.^272^

### Oxeiptosis

Oxeiptosis is an ROS-induced, caspase-independent form of RCD mediated by the KEAP1 (Kelch-like ECH-associated protein 1)-PGAM5 (PGAM family member 5, mitochondrial serine/threonine protein phosphatase)-AIFM1 axis (Fig. 6b).^274^ It was originally described in epithelial and immune cell models exposed to high oxidative stress, including ozone and influenza-associated ROS production.^274^ Mechanistically, elevated ROS levels disrupt the interaction between KEAP1 and PGAM5, permitting PGAM5-dependent activation of AIFM1-associated death signaling.^274^ Compared with ferroptosis, oxeiptosis appears to depend more on ROS-triggered signaling than on extensive lipid peroxidation or overt membrane damage. In mouse models of ozone exposure or influenza infection, loss of PGAM5 reduces oxidative cell death but increases inflammatory cytokine production, suggesting that oxeiptosis may limit inflammatory responses under some conditions.^274^ Oxeiptosis-associated signaling has also been reported in models of oxidative stress-related tissue injury, including noise-induced hearing loss, although its relationship to other ROS-dependent death pathways remains unclear.^275^ The broader physiological relevance of oxeiptosis and its distinction from other ROS-associated death programs remain to be determined.

### Alkaliptosis

Alkaliptosis is a pH-dependent form of RCD associated with intracellular alkalinization (Fig. 6c). It was initially described in pancreatic cancer models treated with the small molecule JTC801, in which sustained elevation of intracellular pH preceded cell death and occurred independently of classical apoptosis.^276^ Mechanistically, JTC801-induced alkaliptosis involves nuclear factor kappa-light-chain-enhancer of activated B cells (NF-κB)-dependent suppression of carbonic anhydrase 9 (CA9), which leads to impaired proton buffering and disruption of intracellular pH regulation.^276^

Additional studies have linked alkaliptosis to lysosomal proton handling and lipid metabolic regulation. ATPase H^+^-transporting V0 subunit D1 (ATP6V0D1)-dependent lysosomal acidification and signal transducer and activator of transcription 3 (STAT3) signaling influence susceptibility to JTC801-induced alkaliptosis, whereas inhibition of vacuolar H^+^-ATPase activity alters intracellular pH dynamics and cell death sensitivity.^277^ Intracellular alkalinization induced by JTC801 also reduces ER cholesterol levels and activates sterol regulatory element binding transcription factor 2 (SREBF2)-dependent cholesterol biosynthesis programs. Cytochrome P450 family 51 subfamily A member 1 (CYP51A1)-mediated cholesterol metabolism subsequently limits lysosomal cholesterol accumulation and promotes transmembrane protein 175 (TMEM175)-dependent lysosomal proton efflux, thereby suppressing alkaliptotic death.^278^ These findings indicate that alkaliptosis is influenced not only by cytosolic pH changes but also by coordination among lysosomal acidification, membrane lipid composition, and intracellular proton transport.

Intracellular alkalinization may also contribute to cell death in non-malignant cells. In CD8^+^ effector T cells, ammonia generated from glutamine metabolism accumulates during the contraction phase and causes lysosomal alkalinization, impairing lysosomal function and mitophagy, ultimately leading to cell death.^279^

Although the molecular basis of alkaliptosis is less extensively characterized than that of ferroptosis or cuproptosis, this pathway illustrates how disruption of proton balance can influence multiple metabolic systems simultaneously. Intracellular pH directly affects enzyme activity, mitochondrial metabolism, ion transport, and redox regulation, and even modest alkalinization can alter the metabolic state of stressed cells.

### PANoptosis

PANoptosis is a lytic inflammatory cell death pathway in which pyroptotic, apoptotic, and necroptotic signaling modules become functionally integrated through PANoptosome assembly (Fig. 6d).^280^ Unlike pyroptosis, in which inflammasome activation is the dominant organizing principle, PANoptosis emerges when inflammatory and metabolic stress promote the coordinated engagement of multiple death machineries that become functionally interdependent. This concept emerged mainly from infection and cytokine-storm models in which inhibition of a single death pathway failed to fully suppress inflammatory cell death, suggesting that immune cells can engage interconnected death programs under conditions of overwhelming stress.^281–286^ PANoptotic signaling is typically initiated by innate immune sensors and organized through multiprotein PANoptosome complexes, including ZBP1-,^287–290^ AIM2-,^289,291,292^ RIPK1-,^293–296^ NLR family CARD domain containing 5 (NLRC5)-,^282^ and NLR family pyrin domain containing 12 (NLRP12)-associated assemblies.^283,297^ These complexes coordinate the activation of caspases, gasdermins, MLKL, and NINJ1,^42,285,288,298–301^ thereby promoting membrane permeabilization and the release of cytokines, chemokines, and DAMPs.

A defining feature of PANoptosis is that its execution cannot usually be explained by activation of any single canonical death pathway. In contrast to classical pyroptosis, apoptosis, or necroptosis, in which disruption of key signaling or effector components can block cell death, PANoptotic death often persists despite inhibition of individual pathways and is more effectively suppressed only when multiple caspase- and RIPK-dependent modules are targeted simultaneously. Thus, PANoptosis is defined not simply by concurrent activation of different death pathways but by functional interdependence among inflammatory death machineries.

Conceptually, PANoptosis is best understood as an integrated inflammatory stress response rather than a death pathway driven by a single biochemical lesion. Diverse signals, including interferon responses, cytokine amplification, nitric oxide-associated metabolic disruption, itaconate metabolism, heme metabolism, NAD^+^ depletion, and mitochondrial stress responses, can lower the threshold for coordinated activation of multiple death programs.^281–283,292,298,302,303^ Under these conditions, pyroptotic, apoptotic, and necroptotic machineries no longer operate as largely independent pathways but instead become functionally interconnected and mutually reinforcing.

The mechanistic boundaries of PANoptosis are not yet fully resolved. PANoptosome composition varies across stimuli and cell types, and many proposed PANoptotic features overlap with broader inflammatory injury responses. Whether PANoptosis represents a distinct molecular pathway or a context-dependent convergence of multiple inflammatory death programs remains an active area of investigation.^304^

### Cuproptosis

Cuproptosis is a copper-dependent form of RCD closely linked to mitochondrial metabolism. It was initially defined through studies of copper ionophores, particularly elesclomol, which preferentially kill cells with active oxidative phosphorylation.^305^ Mechanistically, copper interacts with lipoylated components of the TCA cycle, including dihydrolipoamide S-acetyltransferase (DLAT) and related mitochondrial enzyme complexes, promoting the aggregation of lipoylated proteins together with the loss of iron-sulfur cluster-containing proteins (Fig. 6e).^305^ Ferredoxin 1 (FDX1) and the mitochondrial lipoylation machinery are key determinants of cuproptotic sensitivity. FDX1 regulates mitochondrial protein lipoylation through interaction with lipoic acid synthetase (LIAS), thereby linking cuproptosis to maintenance of the lipoylated TCA cycle network and oxidative metabolism.^306^ Structural studies have identified conserved residues within the α-helix 3 region of FDX1 as critical for both protein lipoylation and cuproptotic sensitivity.^307^

A defining feature of cuproptosis is its metabolic selectivity. Copper accumulation alone does not fully explain this form of death; toxicity depends on the presence of lipoylated mitochondrial proteins and a metabolic state that permits copper-dependent protein aggregation and proteotoxic stress. This distinguishes cuproptosis from nonspecific heavy-metal toxicity and highlights the importance of mitochondrial metabolic state in determining susceptibility to copper-induced cell death.

Intracellular copper-buffering systems influence susceptibility to cuproptosis. In pancreatic ductal adenocarcinoma models, NFE2-like BZIP transcription factor 2 (NFE2L2, also known as NRF2)-dependent expression of solute carrier family 25 member 39 (SLC25A39, also known as CGI-69) promotes mitochondrial GSH uptake, thereby suppressing cuproptotic stress through buffering of labile copper and maintenance of mitochondrial copper homeostasis.^308^ Increased metallothionein expression downstream of HIF1A activation or BACH transcriptional regulator 1 (BACH1) loss can also reduce susceptibility to cuproptosis in some cancer models.^309,310^ Conversely, MYC (MYC proto-oncogene, BHLH transcription factor)-dependent upregulation of DLAT and isocitrate dehydrogenase (NADP^+^) 1 (IDH1) is associated with increased sensitivity to the copper ionophore elesclomol, whereas suppression of oxidative metabolism and protein lipoylation reduces cuproptotic susceptibility.^311^

Overall, cuproptosis is best understood as a failure of mitochondrial copper homeostasis and protein quality control in metabolically susceptible cells rather than as generalized copper toxicity. Although cuproptosis was originally proposed as mechanistically distinct from ferroptosis, several studies suggest partial metabolic overlap between these pathways. For example, low concentrations of heme can destabilize mitochondrial complex IV, promote copper release, and induce cuproptosis-like death in acute myeloid leukemia models,^312^ and excess copper has been reported to enhance ferroptotic sensitivity through destabilization of GPX4.^313^ Together, these findings indicate that metal homeostasis, mitochondrial metabolism, and lipid antioxidant systems can intersect under certain stress conditions, although the mechanistic relationship between cuproptosis and ferroptosis remains to be resolved.

### Disulfidptosis

Disulfidptosis is a form of RCD caused by disulfide stress in cells with high cystine uptake and limited glucose metabolism (Fig. 6f). It was initially identified in SLC7A11-high cancer cells, where cystine import supports GSH synthesis under nutrient-replete conditions but becomes toxic during glucose starvation.^314^

This pathway is closely linked to NADPH-dependent thiol reduction. Under glucose limitation, SLC7A11-high cells continue to import cystine but lose the reducing capacity required to convert cystine to cysteine, leading to intracellular disulfide accumulation rather than lipid peroxide accumulation. A defining feature of disulfidptosis is collapse of the actin cytoskeleton. Glucose starvation induces aberrant disulfide bonding in actin-associated proteins, followed by F-actin collapse and rapid cell death.^314^

Disulfidptosis differs from ferroptosis in that the primary lesion is thiol imbalance and disulfide stress rather than phospholipid peroxidation. At the same time, this pathway illustrates a metabolic trade-off: cellular adaptations that support antioxidant defense under nutrient-replete conditions can become liabilities when reducing equivalents are limited.

More recent studies have suggested that disulfide stress can also arise through disruption of the thioredoxin system. In glioblastoma models, inhibition of thioredoxin reductase 1 (TXNRD1, also known as TRXR1) induces disulfide stress-associated death accompanied by actin cytoskeletal collapse, particularly under glucose-limited conditions.^315^ Disulfidptosis-like signaling is linked to exhausted intratumoral CD8^+^ T cells, in which lactate dehydrogenase B (LDHB)-dependent inhibition of glucose-6-phosphate dehydrogenase (G6PD) reduces NADPH availability and promotes disulfide stress.^316^

Much of the mechanistic evidence for disulfidptosis derives from glucose-starved SLC7A11-high cancer models, and the broader physiological relevance of these findings remains to be determined.

## Recently proposed forms of RCD

### Triaptosis

**Fig. 7:**
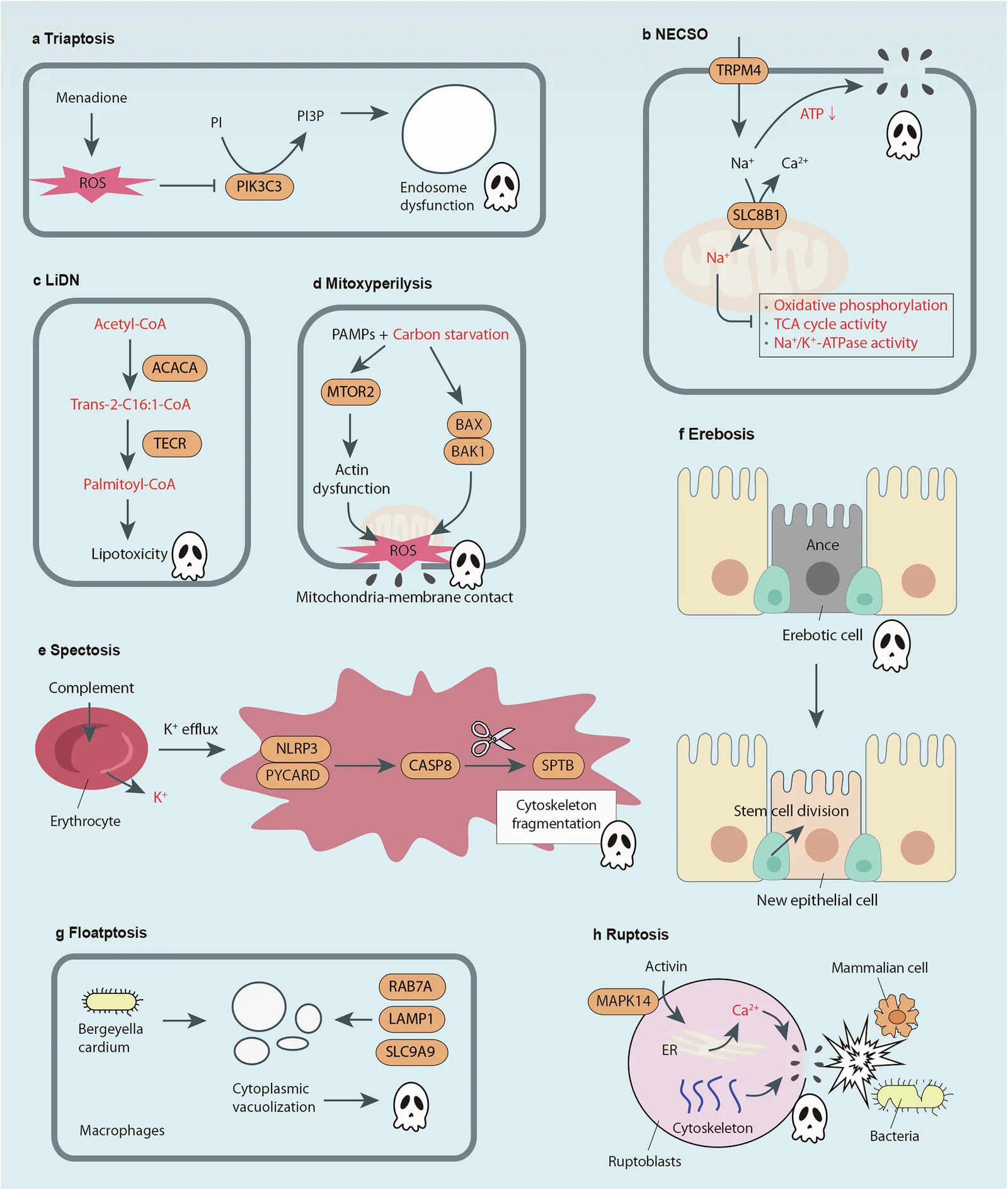
Metabolic regulation of triaptosis, NECSO, LiDN, mitoxyperilysis, spectosis, erebosis, floatptosis, and ruptosis. **a** Triaptosis is associated with oxidative stress-induced disruption of endosomal homeostasis. Oxidative inactivation of phosphatidylinositol 3-kinase catalytic subunit type 3 (PIK3C3/VPS34) depletes phosphatidylinositol 3-phosphate (PI3P), resulting in defective endosomal maturation, impaired membrane trafficking, vesicular accumulation, and cell death. **b** Necrosis by sodium overload (NECSO) is a regulated necrotic cell death pathway driven by sustained intracellular Na^+^ accumulation. Persistent Na^+^ influx, including that mediated by transient receptor potential cation channel subfamily M member 4 (TRPM4), disrupts ionic homeostasis and induces osmotic swelling, membrane damage, and cell lysis. Maintenance of Na^+^ gradients requires continuous ATP consumption via Na^+^/K^+^-ATPase activity. As ATP levels decline, ion pump failure further exacerbates Na^+^ accumulation and osmotic stress. Na^+^ overload also affects mitochondrial function through Na^+^/Ca^2+^ exchanger solute carrier family 8 member B1 (SLC8B1/NCLX)-mediated Na^+^/Ca^2+^ exchange, impairing oxidative phosphorylation and tricarboxylic acid (TCA) cycle activity. **c** Lipid-dependent necrosis (LiDN) is a non-apoptotic cell death program dependent on the lipid metabolic enzyme trans-2,3-enoyl-CoA reductase (TECR). Acetyl-CoA carboxylase alpha (ACACA)-driven fatty acid synthesis and TECR-dependent metabolism converge on palmitoyl-CoA production, which acts as a key determinant of LiDN susceptibility. **d** Mitoxyperilysis is a proposed oxidative cell death process characterized by stress-induced redistribution of mitochondria toward the plasma membrane. Localized mitochondrial ROS production promotes lipid peroxidation and membrane destabilization. Combined pathogen-associated molecular patterns (PAMPs) and carbon starvation signals activate mechanistic target of rapamycin complex 2 (MTOR2)-dependent cytoskeletal dysfunction together with BCL2-associated X, apoptosis regulator (BAX)- and BCL2 antagonist/killer 1 (BAK1)-mediated mitochondrial stress, facilitating mitochondrial clustering and membrane damage. **e** Spectosis is an inflammatory lytic erythrocyte death pathway triggered by complement activation. It involves potassium (K^+^) efflux, activation of the miniNLRP3-PYCARD-CASP8 signaling axis, and cleavage of the membrane cytoskeletal protein spectrin beta, erythrocytic (SPTB). **f** Erebosis is a form of homeostatic cell elimination identified in enterocytes of the adult *Drosophila* intestine. Erebotic cells progressively lose cellular structures and metabolic activity while accumulating the angiotensin-converting enzyme homolog Ance, a characteristic marker of this process. Following cell elimination, intestinal stem cells proliferate and differentiate into new enterocytes, thereby replenishing the epithelium and maintaining tissue homeostasis. **g** Floatptosis is a vacuolization-associated cell death process identified in macrophages infected with a variant strain of *Bergeyella cardium*. It is characterized by extensive RAB7- and lysosome-associated membrane protein 1 (LAMP1)-positive late endosomal-lysosomal vacuolization, with solute carrier family 9 member A9 (SLC9A9) promoting vacuole fusion and expansion. **h** Ruptosis is an explosive form of cell death identified in activin-responsive glandular cells termed ruptoblasts. Excessive activin signaling induces mitogen-activated protein kinase 14 (MAPK14/p38)-dependent activation, triggering release of ER-derived calcium, actomyosin-mediated signal amplification, and catastrophic plasma membrane rupture. Ruptosis results in the release of diffusible cytotoxic factors capable of eliminating neighboring cells and invading microorganisms.

Triaptosis is associated with oxidative stress-induced disruption of endosomal homeostasis.^317^ It was initially identified in cancer cells exposed to the vitamin K precursor menadione (vitamin K3), which induces intracellular vacuolization accompanied by extensive defects in endosomal trafficking (Fig. 7a).^318^ Mechanistically, oxidation and inactivation of phosphatidylinositol 3-kinase catalytic subunit type 3 (PIK3C3, also known as VPS34) depletes phosphatidylinositol 3-phosphate (PI3P), a key regulator of endosomal maturation and membrane trafficking.^318^ The resulting collapse of PI3P-dependent endosomal function leads to vesicular accumulation, impaired intracellular sorting, and cell death.

Available evidence links triaptosis primarily to endosomal dysfunction rather than to a dedicated execution machinery. Although endosomal trafficking is closely connected to nutrient uptake, receptor recycling, and membrane turnover, the physiological relevance of triaptosis and its relationship to other vacuolization-associated death processes remain to be clarified. Nevertheless, triaptosis illustrates how disruption of phosphoinositide-dependent membrane organization and vesicular trafficking can contribute to cell death.

### Necrosis by sodium overload

Necrosis by sodium overload (NECSO) differs from signaling-driven forms of regulated necrosis in that sodium overload itself serves as the initiating metabolic lesion. It is driven by sustained intracellular Na^+^ accumulation (Fig. 7b).^319^ Persistent Na^+^ influx, particularly during prolonged activation of transient receptor potential cation channel subfamily M member 4 (TRPM4), disrupts ionic homeostasis and promotes osmotic swelling, membrane damage, and eventual cell lysis.^319^

NECSO is linked to cellular energy balance because maintenance of intracellular Na^+^ gradients requires continuous ATP consumption through Na^+^/K^+^-ATPase activity. Sustained Na^+^ influx therefore increases the energetic demand for preservation of ion homeostasis. As ATP availability declines, ion pump function becomes impaired, leading to further Na^+^ accumulation, osmotic stress, and membrane destabilization.^319^

Sodium overload also affects mitochondrial metabolism. Acute intracellular Na^+^ elevation in perfused hearts disrupts the TCA cycle and electron transport chain, lowers ATP energetic efficiency, and induces pseudohypoxic signaling through mechanisms involving mitochondrial Na^+^/Ca^2+^ exchange and oxidative stress.^320^ TRPM4-mediated Na^+^ influx increases mitochondrial Na^+^ accumulation and alters mitochondrial Ca^2+^ handling through the Na^+^/Ca^2+^ exchanger solute carrier family 8 member B1 (SLC8B1, also known as NCLX), suppressing oxidative phosphorylation and TCA cycle activity.^321^ Reduced mitochondrial energy production further compromises ion homeostasis, linking ionic imbalance to escalating energetic failure.

Although NECSO is less well characterized than apoptosis or necroptosis, it highlights a metabolic principle: maintenance of electrochemical gradients requires continuous energy expenditure. When ATP production can no longer sustain ionic balance, energetic dysfunction and membrane injury become mutually reinforcing, ultimately leading to irreversible cell lysis.

### Lipid-dependent necrosis

Disruption of lipid homeostasis can trigger forms of RCD that are mechanistically distinct from ferroptosis. Caspase-independent lethal 56 (CIL56) was initially identified as a small molecule capable of inducing ferroptosis at low doses and a non-ferroptotic form of cell death at higher doses.^322^ The cell death induced by high-dose CIL56 requires acetyl-CoA carboxylase alpha (ACACA, also known as ACC1)-dependent de novo fatty acid synthesis and is modulated by fatty acyl-CoA metabolism, protein S-acylation, and membrane trafficking pathways.^323^ More recently, the clinical-stage compound tegavivint was shown to induce a related non-apoptotic death program termed lipid-dependent necrosis (LiDN), which is distinct from apoptosis, ferroptosis, necroptosis, and pyroptosis and requires the lipid metabolic enzyme trans-2,3-enoyl-CoA reductase (TECR) (Fig. 7c).^324^ ACACA-dependent fatty acid synthesis and TECR-dependent metabolism converge on the generation of palmitate and palmitoyl-CoA, identifying saturated fatty acid metabolism as a key determinant of LiDN susceptibility.^324^ In contrast to ferroptosis, high-dose CIL56- or tegavivint-induced LiDN is associated with perturbations in saturated fatty acid metabolism rather than phospholipid peroxidation. Although the downstream execution mechanisms are unclear, LiDN expands the spectrum of lipid-dependent cell death processes and highlights lipid homeostasis as an important determinant of cell fate.

### Mitoxyperilysis

Mitoxyperilysis is a recently proposed form of oxidative cell death characterized by stress-induced redistribution of mitochondria toward the plasma membrane, where mitochondrial ROS damage local membrane lipids, leading to the formation of ROS microdomains that compromise the barrier function of the cell membrane (Fig. 7d).^284^ These findings raise the possibility that the subcellular distribution of oxidative stress may influence cell death independently of overall ROS abundance. In the original model, a combination of pathogen-associated molecular patterns (PAMPs) and carbon starvation induced GSH depletion, leading to mechanistic target of rapamycin complex 2 (MTOR2)-dependent actin dysfunction and BAX- and BAK1-mediated mitochondrial oxidative stress.^284^ Because mitoxyperilysis has not been fully characterized, its mechanistic boundaries relative to other ROS-associated death programs remain uncertain. Nevertheless, this concept suggests that oxidative injury may be spatially organized within cells and that organelle positioning can influence how redox stress is transmitted during cell death.

### Spectosis

Spectosis is a lytic erythrocyte death process triggered by complement activation and associated with K⁺ efflux, activation of a miniNLRP3-PYCARD (PYD and CARD domain containing, also known as ASC)-CASP8 signaling axis, and SPTB (spectrin beta, erythrocytic) cleavage (Fig. 7e).^325^ Thus, complement-mediated hemolysis may involve regulated intracellular responses in addition to direct membrane injury caused by the membrane attack complex.^325^ Because mature red blood cells lack nuclei and mitochondria, spectosis appears to arise primarily from disruption of membrane-cytoskeletal integrity and glycolytic-redox balance rather than from classical organelle stress responses. In this setting, erythrocyte survival depends largely on maintenance of ATP production, antioxidant defenses, and spectrin organization. However, spectosis remains to be fully characterized, and its relationship to eryptosis and other forms of inflammatory hemolysis is unresolved.

### Erebosis

Erebosis is a proposed form of homeostatic cell elimination described in enterocytes of the adult *Drosophila* intestine. Erebotic cells exhibit loss of cytoplasmic proteins, organelles, and cellular structures, accompanied by a gradual decline in cellular function rather than acute execution of a canonical death program (Fig. 7f).^326^ Unlike apoptosis, necrosis, or autophagy-dependent death, erebosis does not appear to require established cell death machinery and has been interpreted as a process of cellular decommissioning during epithelial turnover. From a metabolic perspective, progressive loss of mitochondrial and cytoplasmic content suggests a gradual decline in biosynthetic and metabolic capacity.

However, erebosis remains poorly characterized, with limited mechanistic insight and little evidence for conservation beyond the *Drosophila* intestine. Angiotensin-converting enzyme 2 (ACE2)-positive degenerating neurons have been reported in aged brains and those with Alzheimer disease and have been proposed to exhibit features reminiscent of erebosis, but there is not yet direct evidence for a conserved mammalian erebosis program.^327^ Erebosis is therefore best interpreted as a model of homeostatic cell elimination that expands our understanding of the metabolic regulation of cell death.

### Floatptosis

Floatptosis is a vacuolization-associated cell death process identified in macrophages infected with a variant strain of *Bergeyella cardium* (BCV).^328^ It is characterized by extensive cytoplasmic vacuolization involving RAB7A- and lysosomal-associated membrane protein 1 (LAMP1)-positive late endosomal-lysosomal compartments and can be triggered by BCV-derived outer membrane vesicles or transfected β-barrel-like bacterial proteins (Fig. 7g).^328^ Solute carrier family 9 member A9 (SLC9A9) promotes vacuole fusion during this process, whereas amiloride suppresses vacuolization and enhances host resistance to BCV infection.^328^

Floatptosis appears to differ from apoptosis, necroptosis, ferroptosis, and autophagy-dependent death, although limited apoptosis-like cell death may occur secondarily in infected cultures. Because evidence for this process is largely restricted to the original infection model, the mechanisms that underlie floatptosis and its broader relevance to host-pathogen interactions remain uncertain. Nevertheless, the phenotype provides further support for the idea that disruption of endolysosomal organization and membrane trafficking can culminate in vacuolization-associated cell death.

### Ruptosis

A recent study identified an explosive form of RCD termed ruptosis in a previously unrecognized glandular cell type (ruptoblasts) of regenerative planarian flatworms.^329^ Ruptosis is triggered by activin, a member of the transforming growth factor beta 1 (TGFB1, also known as TGF-β) superfamily that functions as both a hormone and an inflammatory cytokine. Excessive activin signaling induces mitogen-activated protein kinase 14 (MAPK14, also known as p38)-dependent activation of ruptoblasts, leading to rapid release of ER-derived calcium, actomyosin-dependent signal amplification, and catastrophic plasma membrane rupture (Fig. 7h).^329^ This process results in the release of potent diffusible cytotoxic factors capable of eliminating neighboring cells and bacteria.^329^ These findings suggest that activin functions as a local danger signal that links tissue surveillance with immune defense. Ruptosis is mechanistically distinct from apoptosis, necroptosis, pyroptosis, and ferroptosis, but whether similar pathways exist in mammals and contribute to human disease remains unclear.

## Endogenous regulatory circuits that determine metabolic death thresholds

Metabolic stress does not inevitably lead to cell death. Cells possess multiple adaptive systems that help to sustain energy production, redox balance, and organelle function under changing environmental conditions. Whether a metabolic disturbance is reversible or progresses to irreversible injury depends not only on the severity of the insult but also on the effectiveness of these protective mechanisms. Cell death, therefore, arises not simply from metabolic stress itself, but from the failure of compensatory mechanisms to preserve metabolic homeostasis and cellular function.

Many of these regulatory networks are highly interconnected. Nutrient-sensing pathways influence antioxidant defenses; redox status affects gene expression and chromatin organization; and organelle quality-control mechanisms help preserve metabolic function during stress. As perturbations persist, however, compensatory responses may become insufficient or begin to impose additional physiological burdens. The point at which protective mechanisms can no longer offset ongoing metabolic disruption often determines whether cells recover, enter a dysfunctional state, or progress toward cell death.

### Nutrient sensing and metabolic adaptation

Cells continuously monitor nutrient availability and energetic status to coordinate growth, biosynthesis, and stress responses, with AMPK and MTOR forming a central regulatory axis. AMPK is activated during energetic stress and promotes ATP conservation through inhibition of anabolic metabolism, stimulation of autophagy, and preservation of mitochondrial function. By contrast, MTOR integrates amino acid, growth factor, and energy signals to support protein synthesis, lipid synthesis, nucleotide production, and cell growth. These pathways influence not only adaptation to metabolic stress but also the point at which metabolic dysfunction progresses to cell death.

The effects of AMPK on cell death depend on the nature and duration of the metabolic stress and on the downstream pathways engaged. AMPK engages multiple downstream programs that regulate lipid metabolism, autophagy, mitochondrial function, redox homeostasis, and stress responses. Consequently, AMPK activation can either increase or decrease susceptibility to cell death, depending on which downstream responses predominate in a given setting. In apoptosis, AMPK links energetic imbalance to mitochondrial responses, tumor protein p53 (TP53, also known as p53) signaling, and apoptotic susceptibility.^330–332^ In necroptosis, AMPK activation limits necroptotic signaling through mitochondrial quality-control and mitophagy-related pathways, although RIPK3-dependent metabolic remodeling also activates AMPK during early necroptotic responses.^38,127,128^ In pyroptosis, AMPK suppresses NLRP3 inflammasome activation by promoting autophagy, preserving mitochondrial homeostasis, and limiting ROS accumulation.^333,334^

AMPK also participates in metabolically defined death pathways. In ferroptosis, AMPK suppresses lipid peroxide accumulation by inhibiting ACACA-dependent fatty acid synthesis and reducing the abundance of peroxidation-prone phospholipids.^335^ Under prolonged nutrient stress, however, selective autophagy, increased iron availability, and lipid droplet remodeling promote ferroptosis when antioxidant defenses are overwhelmed.^335–337^ In disulfidptosis, glucose deprivation limits NADPH availability in SLC7A11-high cells, preventing the efficient reduction of imported cystine and promoting disulfide stress, cytoskeletal collapse, and cell death, although direct regulation by AMPK remains uncertain.^338^ AMPK is also linked to entosis, in which glucose starvation generates metabolically stressed AMPK-high “loser” cells that are preferentially internalized and eliminated by neighboring cells.^236^

MTOR has complementary effects on cellular responses to metabolic stress and cell death. By sustaining anabolic growth, MTOR increases cellular dependence on nutrient supply, mitochondrial function, and redox capacity. In apoptosis, hyperactive MTOR signaling promotes survival through protein synthesis and the transmission of trophic signals by growth factors,^339,340^ but it also increases susceptibility to energetic collapse when nutrient supply fails to meet anabolic demand.^341^ MTOR suppresses autophagy,^342^ thereby counteracting autophagy-dependent and lysosome-associated death.^343^ It also influences ferroptosis through the regulation of lipid metabolism, autophagy, and ferritinophagy.^344,345^ In necroptosis, MTOR signaling promotes the activation of RIPK3 by limiting its autophagic degradation, establishing an MTOR-RIPK3 axis that contributes to inflammatory tissue injury.^346^ MTOR also affects inflammasome activity through regulation of cellular metabolism, autophagy, and lysosomal function.

There is limited evidence linking AMPK and MTOR to several less extensively studied death pathways. During cuproptosis, copper-induced mitochondrial dysfunction causes ATP depletion and AMPK activation, promoting HMGB1 release and inflammatory signaling, whereas cuproptosis-associated DLAT signaling activates mTOR-dependent pathways that regulate autophagy and therapeutic responses.^347,348^ In parthanatos, PARP1-dependent ATP depletion activates AMPK, which suppresses BCL2-like 11 (BCL2L11, also known as BIM)-dependent cell death, and mTOR promotes parthanatos-like injury through the SIRT1 (sirtuin 1)-PARP1-AIFM1 signaling axis in photoreceptor cells.^349,350^ AMPK activation also accompanies Jaspine B-induced methuosis, although its mechanistic contribution remains unclear.^351^

In conclusion, AMPK and MTOR operate upstream of multiple RCD pathways and help to determine the threshold at which metabolic stress becomes lethal. Whether cells adapt to nutrient limitation or progress to irreversible injury depends in part on the effectiveness of these nutrient-sensing systems in sustaining energy balance, biosynthetic coordination, and stress adaptation.

### Redox buffering and metabolic resilience

The ability to maintain redox balance is a major determinant of metabolic resilience. ROS are continuously generated during mitochondrial respiration, lipid metabolism, and inflammatory signaling, but cell survival depends less on absolute oxidant production than on the capacity of endogenous reducing systems to maintain redox balance. Whether oxidative stress remains adaptive or progresses to cell death is therefore determined by the ability of cells to preserve reducing power, repair oxidized biomolecules, and sustain compartmentalized redox control.

NADPH occupies a central position in these defenses because it supplies reducing equivalents for GSH regeneration, thioredoxin-dependent reduction, and multiple antioxidant reactions.^352^ Pathways that generate NADPH, including the pentose phosphate pathway, one-carbon metabolism, and malic enzyme activity, strongly influence susceptibility to metabolic stress. When reducing capacity falls below demand, oxidative damage accumulates, and death thresholds are lowered.

This principle is evident in ferroptosis and disulfidptosis. In ferroptosis, inadequate reducing capacity impairs the detoxification of phospholipid hydroperoxides and favors lipid peroxidation.^14^ In disulfidptosis, glucose deprivation limits NADPH production in SLC7A11-high cells, preventing the efficient reduction of imported cystine and promoting disulfide stress.^314^ In both settings, cell death reflects the failure of redox buffering systems rather than oxidant accumulation alone.

The importance of metabolic reducing capacity extends beyond these pathways. In parthanatos, excessive PARP1 activation consumes large amounts of NAD^+^, leading to secondary ATP depletion and energetic collapse.^264^ Cell survival, therefore, depends on both the extent of DNA damage and the ability to preserve nucleotide pools and bioenergetic function. Similarly, cuproptosis is accompanied by profound mitochondrial dysfunction and ATP depletion, indicating that susceptibility to copper-induced death is influenced by the capacity to maintain mitochondrial metabolic and redox functions under stress.^353^

GSH, thioredoxin, and related thiol-dependent pathways constitute the major intracellular redox buffering network. These systems not only limit oxidative damage but also preserve protein folding, cytoskeletal integrity, and organelle function. Their activity is tightly coupled to nutrient availability and cellular metabolism, making redox regulation an active metabolic process rather than a passive antioxidant defense mechanism.

Redox adaptation can itself create metabolic dependencies. Cells chronically exposed to oxidative stress frequently increase antioxidant capacity and become reliant on sustained NADPH production, cysteine availability, and thiol metabolism. Cancer cells provide a well-studied example of this phenomenon. Enhanced antioxidant programs support survival under metabolic stress but simultaneously create vulnerabilities when reducing capacity becomes limiting.^354^ This distinction is particularly evident when comparing acute and chronic adaptation. Short-term activation of antioxidant pathways generally increases stress tolerance and restores redox balance. By contrast, sustained antioxidant reprogramming tends to increase dependence on cysteine availability, NADPH production, and thiol metabolism.^355–359^ Adaptations that initially support survival can therefore generate new metabolic liabilities that become apparent when nutrient supply or reducing capacity becomes limiting.

Oxidative cell death is determined by the balance between oxidative burden and buffering capacity.^360^ However, the sources of oxidative stress and the antioxidant systems that constrain it are highly dynamic and spatially organized, making it difficult to define their relative contributions. Disruption of redox balance is not limited to oxidative stress, as excessive reducing capacity can also become detrimental and contribute to reductive cell death.^361^ For example, impaired de novo fatty acid synthesis sensitizes cells to NADPH-driven reductive stress by limiting the consumption of excess reducing equivalents, resulting in catastrophic accumulation of NADH and GSH-dependent reducing power and accelerated cell death.^362^ The development of improved approaches for measuring compartment-specific redox activity will be essential for understanding how metabolic stress crosses the threshold to irreversible cell death.

### Transcriptional and chromatin-level coupling between metabolism and cell fate

Metabolic adaptation includes more than acute biochemical responses. When metabolic stress persists, cells engage transcriptional and chromatin-based programs that reshape nutrient utilization, redox balance, organelle function, and stress tolerance. These responses can maintain survival under adverse conditions, but they can also alter the threshold at which metabolic stress progresses to irreversible injury and cell death.

Several stress-responsive transcription factors act as major interfaces between metabolism and cell fate. NFE2L2 coordinates antioxidant defense by inducing genes involved in GSH synthesis, NADPH production, and iron metabolism. This response strengthens antioxidant defenses and reduces susceptibility to oxidative stress-associated cell death under many conditions.^308,363,364^ However, sustained NFE2L2 activation can also increase dependence on cystine uptake, NADPH generation, and thiol metabolism, creating metabolic vulnerabilities that become evident when nutrient supply or reducing capacity is limited.^365^

The integrated stress response downstream of eukaryotic translation factor 2A (EIF2A, also known as eIF2α) phosphorylation provides another example. Amino acid deprivation, proteotoxic stress, and organelle dysfunction stimulate autophagy^366^ and activate activating transcription factor 4 (ATF4)-dependent transcriptional programs that promote amino acid transport, redox control, and metabolic adaptation.^367,368^ These responses help to re-establish cellular function during transient stress, but persistent ATF4 activation often reflects unresolved metabolic dysfunction and is associated with a loss of cellular fitness.

TP53 occupies a distinct position within these networks because it integrates metabolic stress with decisions governing adaptation, survival, and death. In response to nutrient limitation, oxidative stress, or DNA damage, TP53 regulates genes involved in glucose metabolism, mitochondrial function, redox homeostasis, and cell fate determination.^369^ Depending on the cellular context, TP53 can promote metabolic adaptation and stress tolerance or facilitate apoptosis and ferroptosis through transcriptional programs that are engaged when cellular damage exceeds the capacity for recovery.^370–372^ Emerging evidence also links TP53 to the regulation of cuproptosis susceptibility.^373^ TP53 exerts both pro- and anti-ferroptotic effects, highlighting its role as a context-dependent regulator of metabolic stress responses.^370,371^ TP53 therefore functions as an important checkpoint linking metabolic stress to cell fate commitment.^374^

Hypoxia-inducible factors (HIFs) reveal how transcriptional adaptation influences death susceptibility. By promoting glycolysis, suppressing mitochondrial respiration, and remodeling nutrient utilization, HIF signaling supports survival during oxygen limitation.^375,376^ At the same time, these metabolic changes reshape susceptibility to oxidative stress, apoptosis, ferroptosis, and cuproptosis, demonstrating that adaptive transcriptional programs can modify the response to subsequent insults.^310,377^

Metabolism also influences cell fate through direct effects on chromatin regulation. Several metabolites serve as substrates or cofactors for chromatin-modifying enzymes. Acetyl-CoA supports histone acetylation, S-adenosylmethionine provides methyl groups for DNA and histone methylation, α-ketoglutarate regulates dioxygenases involved in chromatin remodeling, and NAD^+^ controls the activity of sirtuins and other chromatin-associated enzymes.^378^ Changes in metabolite availability therefore influence transcriptional accessibility and the expression of stress-responsive genes.

Under prolonged metabolic stress, these transcriptional and epigenetic responses can become stabilized. In cancer, persistent metabolic rewiring reshapes chromatin landscapes that influence oxidative stress tolerance, therapy resistance, and susceptibility to cell death. Similar processes are observed in chronic inflammatory and degenerative disorders, where sustained metabolic dysfunction is accompanied by stable transcriptional programs associated with mitochondrial impairment, cytokine production, and tissue injury.

Transcriptional adaptation is therefore not inherently protective. Programs that initially support metabolic adaptation can, over time, reinforce specific metabolic dependencies and alter susceptibility to cell death. Under prolonged stress, these transcriptional and epigenetic states may become stabilized, reshaping cellular identity and stress tolerance. A central unresolved question is how metabolic and epigenetic signals are translated into selective cell-fate decisions. Stress-responsive transcription factors often regulate both adaptive and pro-death programs, whereas chromatin states influence which target genes remain accessible under metabolic stress. Defining the transcriptional and epigenetic targets that govern death-pathway selection under different metabolic conditions will therefore be essential for understanding how similar metabolic perturbations give rise to distinct cellular outcomes.

### Organelle coordination and metabolic stress buffering

Metabolic stress is rarely confined to a single organelle. Mitochondria, lysosomes, the ER, and vesicular trafficking systems continuously exchange metabolites, ions, and signaling molecules to support cellular function under changing conditions. These interactions help to limit metabolic perturbations and promote stress adaptation. As stress intensifies or persists, however, disruption of organelle coordination can convert localized dysfunction into a propagating process that culminates in irreversible cellular injury and death.

Communication between the ER and the mitochondria provides a well-established example of this principle. Contact sites between these organelles regulate calcium transfer, lipid exchange, and mitochondrial bioenergetics. Under physiological conditions, controlled calcium flux supports ATP production and metabolic flexibility. During sustained stress, excessive calcium transfer promotes mitochondrial overload, oxidative stress, and permeability transition. Disruption of ER–mitochondrion communication is therefore frequently associated with apoptosis and other forms of mitochondrial injury.^149,379–381^

The lysosome-mitochondrion axis represents another important mechanism for maintenance of metabolic function. Lysosomes support mitochondrial activity through nutrient recycling, mitophagy, and iron regulation. During transient stress, these processes facilitate recovery by removing damaged organelles and redistributing intracellular resources. However, lysosomal dysfunction can promote the accumulation of damaged mitochondria, impair bioenergetic recovery, and increase oxidative stress. This interaction is particularly evident in ferroptosis, during which ferritinophagy and lysosomal iron release expand labile iron pools and facilitate lipid peroxidation under conditions of impaired antioxidant defense.^150^ Lysosomal damage can also contribute to inflammatory signaling through activation of inflammasome pathways in specific contexts.^382^

Organelle communication also influences the spatial transmission of metabolic stress. Mitochondrial dysfunction can trigger ER stress, alter lysosomal activity, and reshape the intracellular redox balance, and defects in lysosomal degradation further exacerbate mitochondrial injury by limiting organelle turnover and metabolic recycling. Similar principles are observed in cuproptosis, during which mitochondrial proteotoxic stress initiates secondary metabolic disturbances that extend beyond the mitochondrion itself.^305^ Together, these findings demonstrate that metabolic stress is often propagated through interconnected organelle networks.

Vesicular trafficking pathways provide an additional layer of metabolic regulation. Endosomal transport, membrane recycling, and autophagic flux influence nutrient availability, receptor turnover, and compartment identity. Efficient trafficking supports cellular adaptation during stress, whereas defects in membrane trafficking disrupt organelle function and reduce the capacity of the cell to withstand metabolic perturbations. This relationship is apparent in vacuolization-associated death pathways such as methuosis, in which dysregulated macropinosome trafficking overwhelms intracellular membrane systems.^243^

Organelle communication networks therefore function not simply as structural connections but as integrated systems that coordinate metabolic adaptation. Their ability to regulate nutrient recycling, mitochondrial quality control, calcium signaling, and membrane dynamics strongly influences whether metabolic stress remains manageable or progresses toward cell death. A challenge will be to identify the molecular signals that determine when organelle crosstalk shifts from adaptive coordination to progressive dysfunction, thereby committing cells to death.

## Immunological consequences of cell death

The consequences of cell death extend beyond the removal of damaged cells. During cellular disassembly, dying cells release metabolites, DAMPs, cytokines, and oxidized biomolecules that influence the surrounding tissue environment. ATP performs both metabolic and immunological functions, acting as an intracellular energy carrier and an extracellular danger signal, and oxidized phospholipids, lipid peroxidation products, and mitochondrial components released from injured cells can engage innate immune sensing pathways. The immune response to cell death is therefore influenced by both the execution machinery involved and the metabolic composition of the signals released during cellular disassembly.^383,384^

Different forms of RCD generate distinct extracellular environments. Pyroptosis, necroptosis, and PANoptosis are accompanied by the release of IL1B, IL18, HMGB1, ATP, and other inflammatory mediators that promote innate and adaptive immune responses.^385,386^ Ferroptosis is associated with the accumulation of oxidized phospholipids and reactive lipid metabolites that influence immune cell function and inflammatory signaling in a context-dependent manner.^137,387,388^ By contrast, apoptotic cells generally preserve plasma membrane integrity during the early stages of disassembly and preferentially release metabolites linked to anti-inflammatory signaling and tissue repair. These differences indicate that the metabolic alterations that precede cell death contribute to the balance between immune activation, immune resolution, and immune tolerance.

The relationship between metabolism and immunity is further illustrated by immunogenic cell death (ICD), a functional state in which dying cells stimulate antigen-presenting cells and adaptive immune responses. This concept emerged from studies showing that certain forms of therapy-induced apoptosis, including doxorubicin-induced tumor cell death, can elicit protective antitumor immunity.^389^ Hallmarks of ICD include ATP release, calreticulin (CALR) exposure, and HMGB1 secretion, which facilitate dendritic cell recruitment, activation, and antigen presentation.^390–393^ Extracellular GPX4, selectively released from ferroptotic cells, suppresses glycolysis and maturation of dendritic cells through zona pellucida glycoprotein 3 (ZP3)-dependent signaling, thereby limiting the immunogenicity of ferroptotic cell death.^137^ ICD is not a separate execution mechanism and can arise in association with multiple forms of RCD. The acquisition of immunogenic properties depends on the metabolic and inflammatory context in which cell death occurs and the nature of the signals released from the dying cells. Recent studies suggest that cuproptosis may induce a more potent ICD response than apoptosis, necroptosis, or ferroptosis.^137,394^ Although cuproptotic cells release HMGB1 and other common DAMPs, the specific DAMP repertoire underlying the exceptional immunogenicity of cuproptosis remains to be characterized.^395^

## Disease-associated metabolic vulnerabilities and therapeutic opportunities

Disease-associated cell death often reflects disruption of the metabolic systems that sustain cellular function rather than a single molecular defect. Because different tissues operate under distinct energetic, nutritional, and redox demands, susceptibility to cell death frequently arises from selective dependence on specific metabolic programs. Consequently, adaptations that support survival under stress can become sources of vulnerability when metabolic demands exceed the ability of cells to maintain function.

This perspective suggests therapeutic opportunities beyond the targeting of death-execution pathways. Interventions that modulate nutrient utilization, redox regulation, organelle quality control, or stress-adaptive programs may alter susceptibility to cell death and modulate disease progression. Understanding tissue-specific metabolic vulnerabilities may enable the development of new strategies for selectively promoting or preventing cell death while preserving normal tissue function.

### Cancer: exploiting metabolic liabilities

Cancer progression is accompanied by continuous selection for cells capable of surviving fluctuating oxygen availability, nutrient scarcity, and therapeutic pressure. As tumors evolve, they acquire metabolic traits that support growth in hostile microenvironments, but these same adaptations often narrow the range of metabolic states compatible with survival. Therapeutic opportunities often arise from these acquired constraints rather than from the metabolic alterations themselves.

Ferroptosis provides a useful example. Tumors that undergo epithelial-to-mesenchymal transition, acquire drug resistance, or persist in chronically oxidizing environments frequently become reliant on pathways that limit phospholipid peroxidation. Dependence on cystine utilization, GSH metabolism, or GPX4 activity enables these cells to tolerate oxidative stress but also creates selective sensitivity to ferroptosis-inducing interventions.^396,397^ Consequently, ferroptosis-based strategies are being explored as a means to eliminate tumor populations that remain refractory to conventional therapies.

Metabolic specialization also shapes susceptibility to other forms of cell death. Tumors with high mitochondrial activity and sustained flux through the TCA cycle are more sensitive to copper-dependent mitochondrial injury, whereas tumors that rely predominantly on aerobic glycolysis may be comparatively resistant.^305^ Similarly, nutrient-scavenging mechanisms that support survival in resource-poor environments can become exploitable liabilities when nutrient acquisition or intracellular recycling pathways are disrupted.^398–400^

These examples illustrate a broader principle: successful tumor adaptation often narrows metabolic flexibility. Cancer cells frequently operate near the limits of redox control, nutrient utilization, or organelle quality control, leaving them with limited ability to accommodate additional metabolic perturbations. Therapeutic strategies that exploit this restricted flexibility may selectively eliminate malignant cells while sparing normal tissues that retain greater metabolic adaptability.

A major challenge is the marked heterogeneity of tumor metabolism. Distinct regions within the same tumor can differ substantially in oxygenation, nutrient access, mitochondrial activity, and stress responses. Metabolic states may also shift during tumor progression or treatment. Identifying biomarkers that capture these dynamic metabolic states will be essential for translating cell death pathways into effective therapeutic strategies.

### Neurodegeneration: selective vulnerability to metabolic instability

Neurodegenerative diseases illustrate how long-lived cells can become progressively vulnerable to metabolic dysfunction. Unlike proliferating tissues, neurons have limited regenerative capacity and depend on continuous energy production to maintain membrane excitability, synaptic transmission, and axonal transport. As a result, relatively modest disturbances in energy metabolism, redox homeostasis, or organelle function can accumulate over long periods before overt neuronal loss becomes apparent.

A recurring feature across disorders such as Alzheimer disease, Parkinson disease, amyotrophic lateral sclerosis, and Huntington disease is the convergence of multiple metabolic stresses rather than failure of a single pathway. Mitochondrial dysfunction, impaired proteostasis, oxidative stress, and defective intracellular trafficking frequently coexist and reinforce one another. Because neurons rely heavily on oxidative metabolism and long-range organelle transport, they have limited ability to compensate when these systems begin to deteriorate.

Several RCD pathways are implicated in this setting. Ferroptosis has attracted particular attention because the nervous system is enriched in iron and polyunsaturated lipids, creating conditions favorable for lipid peroxidation. Experimental studies have linked ferroptotic signaling to neuronal injury in models of stroke, traumatic brain injury, and several neurodegenerative disorders.^401–404^ Parthanatos represents another example. Excessive PARP1 activation depletes NAD^+^ and ATP, amplifying energetic failure and contributing to neuronal loss in neuroinflammatory and neurodegenerative conditions.^405,406^ Disturbances in metal homeostasis, lysosomal function, and organelle quality-control pathways may further increase neuronal susceptibility to metabolic stress, although their contributions to specific death mechanisms remain to be clarified.^407,408^

Together, these findings demonstrate that neurodegeneration is often driven less by activation of a single death pathway than by progressive deterioration of the systems that sustain metabolic function. Different death programs may predominate in different diseases or disease stages, but they frequently arise from a shared background of mitochondrial dysfunction, impaired redox regulation, and defective organelle quality control. Therapeutic strategies that preserve metabolic function, support organelle maintenance, or improve cellular adaptation to stress may therefore be as important as the direct targeting of individual death pathways.

### Infection and inflammation: immunometabolic amplification of cell death

Infectious and inflammatory diseases illustrate how immune responses reshape cellular metabolism and, in turn, influence susceptibility to cell death. Activation of immune cells is accompanied by marked changes in glucose utilization, mitochondrial activity, and metabolite production that support antimicrobial defense and cytokine secretion. Although these adaptations are essential for host protection, they can also contribute to tissue injury when inflammation becomes excessive or fails to resolve.

Several RCD pathways participate in this process. Pyroptosis is closely linked to immunometabolic signaling, as inflammasome activation is influenced by mitochondrial dysfunction, ROS production, lysosomal damage, and metabolic stress. During infection, pyroptosis promotes pathogen clearance and inflammatory cytokine release, but excessive activation can cause tissue injury and systemic inflammation.^382,409–412^ PANoptosis also illustrates the convergence of inflammatory and metabolic signals,^282,302,413^ whereby pyroptotic, apoptotic, and necroptotic pathways can be activated in parallel in response to severe infection or cytokine exposure.^134,414–416^

Immunometabolic pathways can also limit inflammatory injury. For example, itaconate, a metabolite derived from the TCA cycle, accumulates in activated macrophages and limits inflammatory responses through effects on redox signaling, inflammasome activity, and cellular metabolism.^417–420^ However, excessive or sustained itaconate accumulation can impair mitochondrial function, disrupt redox homeostasis, and amplify inflammatory responses under certain conditions.^292,421–426^ Together, these findings indicate that metabolic rewiring during inflammation can either limit or promote cell death depending on the nature and duration of the response.

Many pathogens modify host metabolism to favor their replication. Viruses frequently increase glycolytic and biosynthetic activity, whereas intracellular bacteria manipulate nutrient acquisition, mitochondrial function, and vesicular trafficking pathways. These alterations reshape cellular stress responses and can influence susceptibility to inflammatory and metabolic forms of cell death.

Inflammatory injury often extends beyond infected cells. Activated macrophages and neutrophils release ROS, nitric oxide, and inflammatory mediators that disrupt mitochondrial function and lipid homeostasis in surrounding tissues. This creates a feed-forward cycle in which tissue injury amplifies inflammation and inflammation further exacerbates metabolic dysfunction.^427^ Ferroptosis has emerged as one contributor to this process in several infectious and inflammatory settings, where iron dysregulation, lipid peroxidation, and oxidative stress promote tissue damage.^428–430^

RCD is therefore not only detrimental during infection. Pyroptosis, NETosis, and, in some contexts, ferroptotic responses can restrict pathogen replication, facilitate immune recognition, and enhance pathogen clearance. The therapeutic challenge is not simply to suppress inflammatory cell death, but to selectively limit tissue injury while preserving the protective functions of host defense pathways.

## Therapeutic strategies for modulating metabolic death thresholds

A metabolism-centered view of cell death shifts the therapeutic focus from death-execution pathways to the metabolic conditions that determine death susceptibility. In many diseases, vulnerability to cell death depends less on the presence of death machinery than on the ability of cells to sustain metabolic function under stress.

Targeting of redox buffering systems provides one example. Ferroptosis induction through inhibition of GPX4, system xc^−^, or GSH synthesis destabilizes antioxidant defenses in tumors that rely on chronic redox adaptation. Conversely, enhancement of antioxidant defenses or suppression of lipid peroxidation may protect neurons and ischemic tissues from oxidative injury. Recent studies on organ transplantation and ischemia-reperfusion injury have shown that ferroptosis inhibitors, including the radical- and iron-trapping compound FXT-001, preserve graft viability and improve tissue recovery under severe metabolic stress.^431^ At the same time, ferroptosis-inducing strategies continue to be developed for cancer therapy. Agents such as N6F11^432^ and fentomycin-1^165^ exploit selective metabolic liabilities in therapy-resistant tumor cell populations, illustrating how modulation of redox buffering and iron metabolism can either increase or decrease death susceptibility depending on the therapeutic objectives.

Manipulation of metal ion homeostasis provides another approach. Iron chelators such as deferoxamine suppress ferroptotic injury in neurodegeneration and ischemia,^401^ whereas the copper ionophore elesclomol exploits mitochondrial copper sensitivity to induce cuproptosis in tumors with active oxidative metabolism.^305^ Because transition metals are integral to mitochondrial function and redox chemistry, changes in metal availability can markedly influence death susceptibility.

Nutrient utilization can also be exploited therapeutically. The restriction of glucose, cystine, or other key nutrients exposes metabolic liabilities in cancer cells, whereas restoration of NAD^+^ metabolism may improve neuronal function and stress tolerance during aging and neurodegeneration. One example is cyst(e)inase, an engineered enzyme that depletes extracellular cysteine and cystine and induces ferroptosis in pancreatic ductal adenocarcinoma and other cystine-dependent tumors, demonstrating how nutrient dependence can be converted into a therapeutic vulnerability.^357,433^ Metabolic cofactors provide another layer of intervention. Vitamin B3-derived NAD^+^ precursors, including nicotinamide riboside and nicotinamide mononucleotide, support mitochondrial function and cellular stress resistance,^434,435^ and lipid-soluble antioxidants such as vitamin E can suppress lipid peroxidation and ferroptotic injury in specific settings.^436,437^ Modulation of autophagy, mitochondrial quality control, and lysosomal recycling provides a complementary strategy for preserving metabolic function before irreversible injury occurs. Compounds such as urolithin A and spermidine enhance autophagic and mitophagic pathways, improve mitochondrial quality control, and are being evaluated in clinical studies for aging-associated disorders.^438–441^ Together, these approaches demonstrate that modulation of metabolic programs can either promote or prevent cell death depending on disease-specific physiological demands.

Because metabolic stress responses are highly interconnected, a combination of approaches may offer particular advantages. In tumors, simultaneous targeting of DNA damage responses and metabolic adaptation can overwhelm compensatory mechanisms. One example is the combination of PARP inhibition with strategies that limit NAD^+^ availability, thereby coupling defects in DNA repair to metabolic stress and energetic dependency.^442^ In inflammatory disease, the combination of metabolic interventions with suppression of pathological immune signaling may reduce tissue injury while preserving host defense.^281^ Targeting multiple nodes within metabolic stress networks may therefore prove more effective than inhibiting individual pathways in isolation.

A major challenge is the tissue- and disease-specific nature of metabolic vulnerabilities. The same pathway may support survival in one tissue but contribute to pathology in another. Effective intervention, therefore, requires an understanding of tissue-specific metabolic organization, stress adaptation, and organelle function.

RCD may reframe therapeutic intervention as the modulation of metabolic states that determine cellular fate, rather than the inhibition of individual death pathways. Cellular outcomes are shaped not only by death signaling itself but also by the capacity of cells to maintain energy production, redox homeostasis, and organelle function under pathological conditions.

## Conclusions and perspectives

Advances in RCD research have revealed that cellular fate is determined not only by dedicated death-execution pathways but also by the metabolic state that governs cellular adaptation to stress. Although individual forms of RCD are defined by distinct molecular execution mechanisms, accumulating evidence indicates that their initiation, progression, and biological consequences are profoundly influenced by bioenergetic status, redox homeostasis, nutrient availability, metal ion metabolism, inflammatory metabolic remodeling, and organelle function. Together, these findings establish metabolism as a fundamental regulator of cellular vulnerability, stress adaptation, and death susceptibility across diverse forms of RCD.

A recurring theme in recent studies is the importance of metabolic resilience. Nutrient-sensing pathways, antioxidant systems, transcriptional adaptation, organelle quality-control networks, and inter-organelle communication continuously preserve cellular homeostasis under fluctuating environmental conditions. Cell death frequently occurs when these adaptive mechanisms become overwhelmed, exhausted, or maladaptively rewired. These shared metabolic principles help to explain why multiple forms of RCD often coexist within the same pathological setting and why similar stresses may activate distinct death programs depending on cell type, tissue context, metabolic state, and disease conditions.

Understanding the metabolic hallmarks of RCD also provides important insights into disease pathogenesis and therapeutic intervention. In cancer, metabolic rewiring creates vulnerabilities that can be exploited therapeutically through synthetic lethal approaches and metabolic targeting. In neurodegenerative disorders, progressive impairment of mitochondrial function, redox homeostasis, and organelle quality control increases susceptibility to irreversible cell loss. In infection and inflammatory diseases, immunometabolic remodeling influences both host defense and collateral tissue injury. Across these settings, susceptibility to cell death reflects the dynamic interplay between metabolic adaptation and the activation of canonical death-execution pathways.

Another message emerging from current research is that metabolic alterations rarely operate in isolation. Individual forms of RCD are frequently influenced by multiple metabolic processes simultaneously, and many metabolic hallmarks are shared among distinct death modalities. Ferroptosis, for example, is shaped by lipid peroxidation, iron metabolism, redox buffering, mitochondrial metabolism, and organelle crosstalk. Likewise, pyroptosis is closely linked to inflammatory metabolic remodeling and mitochondrial dysfunction, whereas cuproptosis reflects interactions among copper homeostasis, mitochondrial metabolism, and proteotoxic stress. These metabolic hallmarks provide complementary biological insights that deepen our understanding of the initiation, regulation, and execution of diverse death programs under physiological and pathological conditions.

Despite remarkable progress, several important questions remain unresolved. A major challenge will be to define how multiple metabolic stress signals are integrated to determine death commitment in specific cellular contexts. The relative contributions of nutrient-sensing pathways, redox-buffering systems, organelle communication networks, metabolic plasticity, and epigenetic regulation are incompletely understood. Likewise, the mechanisms by which similar metabolic perturbations activate different death programs in distinct cell types require further investigation. Addressing these questions will require improved approaches for monitoring metabolic flux, redox dynamics, metabolite compartmentalization, and organelle function with high spatial and temporal resolution in living tissues.

Future therapeutic strategies will likely benefit from integrating metabolic regulation with the molecular mechanisms that execute RCD. Defining tissue-specific metabolic vulnerabilities, adaptive buffering mechanisms, and biomarkers of metabolic state may enable more precise modulation of cell fate in cancer, neurodegeneration, inflammatory diseases, and tissue injury. Rather than replacing existing mechanistic frameworks, continued investigation of the metabolic hallmarks of RCD will provide complementary insights into the mechanisms that govern cellular adaptation and demise, ultimately facilitating the development of more effective therapeutic strategies for preventing pathological cell loss while selectively eliminating diseased cells.
